# Using DNA origami nanorulers as traceable distance measurement standards and nanoscopic benchmark structures

**DOI:** 10.1038/s41598-018-19905-x

**Published:** 2018-01-29

**Authors:** Mario Raab, Ija Jusuk, Julia Molle, Egbert Buhr, Bernd Bodermann, Detlef Bergmann, Harald Bosse, Philip Tinnefeld

**Affiliations:** 1 Institute for Physical & Theoretical Chemistry, and Braunschweig, Integrated Centre of Systems Biology (BRICS) and Laboratory for Emerging Nanometrology (LENA), Braunschweig University of Technology, Rebenring 56, 38106 Braunschweig, Germany; 2 Physikalisch-Technische Bundesanstalt (PTB), Bundesallee 100, 38116 Braunschweig, Germany; 3 Department of Chemistry and Center for NanoScience, Ludwig-Maximilians-Universitaet Muenchen, Butenandtstr, 5–13, 81377 Muenchen, Germany

## Abstract

In recent years, DNA origami nanorulers for superresolution (SR) fluorescence microscopy have been developed from fundamental proof-of-principle experiments to commercially available test structures. The self-assembled nanostructures allow placing a defined number of fluorescent dye molecules in defined geometries in the nanometer range. Besides the unprecedented control over matter on the nanoscale, robust DNA origami nanorulers are reproducibly obtained in high yields. The distances between their fluorescent marks can be easily analysed yielding intermark distance histograms from many identical structures. Thus, DNA origami nanorulers have become excellent reference and training structures for superresolution microscopy. In this work, we go one step further and develop a calibration process for the measured distances between the fluorescent marks on DNA origami nanorulers. The superresolution technique DNA-PAINT is used to achieve nanometrological traceability of nanoruler distances following the guide to the expression of uncertainty in measurement (GUM). We further show two examples how these nanorulers are used to evaluate the performance of TIRF microscopes that are capable of single-molecule localization microscopy (SMLM).

## Introduction

Biology is currently emerging from a descriptive into a quantitative research discipline describing living organisms with quantitative numbers resulting from measurements of concentrations and molecular abundances as well as kinetic rates for metabolic and developmental processes. The determined measurement results can be brought in context with disease, disease probability, and the prognosis of disease progression. Such results can have profound consequences for patient treatment and thus must be very reliable and trustworthy. Therefore, measurement results should be traced back to the International System of Units (SI). It is a major task of national metrology institutes such as the Physikalisch-Technische Bundesanstalt (PTB) in Germany or the National Institute of Standards and Technology (NIST) in the US to provide traceability of measurement results to the SI.

Superresolution microscopy is a field of research that aims at revealing quantitative information on molecule parameters and structural features smaller than the diffraction limit, i.e. smaller than about half the wavelength of light^1–4^. In recent years, especially the resolution capability has been a matter of debate as many competing techniques emerged, culminating in the Nobel Prize in chemistry for superresolution microscopy in 2014^5^. With the vast variety of approaches, techniques and variations of them, the need for fluorescent structures emerged, that would allow a straightforward and fair comparison of the manifold of ideas and superresolution realizations. Samples were needed that offer a defined pattern of fluorescent marks. In addition, these patterns need to have a strict stoichiometric control over the number of fluorescent dye molecules as well as the flexibility to align them in various patterns. For their versatile use, they should also be reproducible, stable and portable to test the same structure on different microscopes and with different methods.

DNA nanotechnology, especially the robust DNA origami technique^6^, has emerged as a LEGO-like toolbox to assemble different objects in precise and pre-defined patterns. In DNA origami, a long scaffold strand of e.g. 7249 nucleotides is folded by hybridizing with about 200 shorter staple strands. Since a wide range of chemical modifications of DNA is available, orthogonal chemistry for placing other objects is easily introduced by replacing specific staple strands with their modified versions. As each staple strand has a well-defined a-priori known position in the final nanostructure, the position of the inserted modification is equally well-known.

In a first combination of single-molecule microscopy and DNA nanotechnology, the ability to place individual fluorescent dyes in a DNA nanostructure was exploited to resolve the nanoscopic distance of about 89 nm between two dye molecules on a rectangular DNA origami^7^ using dSTORM^8^ and Blink Microscopy^9^. The versatility of this approach was demonstrated with a series of nanorulers for diverse microscopy methods from confocal diffraction-limited to 6 nm 2D superresolution^10–15^ and even 3D superresolution^16^. Protocols for production and purification, quality control, handling, immobilization, measurements and data analysis were further developed including many practical aspects such as portability, robustness and reproducibility under different conditions^17^ and led to the first commercial application of the DNA origami technique, i.e. DNA origami nanorulers.

**Figure 1:**
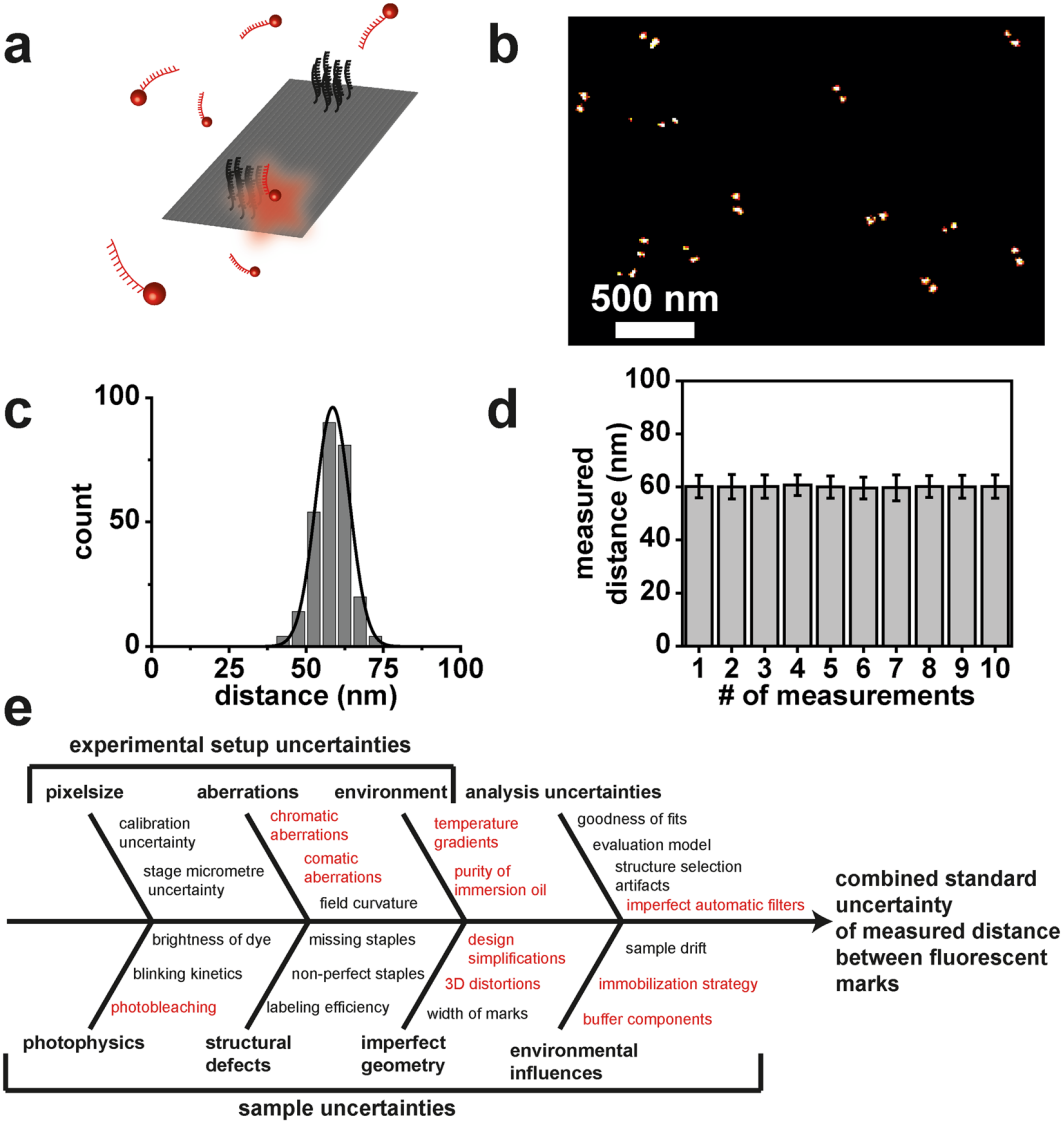
(**a**) Sketch of a rectangular DNA origami nanoruler imaged with DNA PAINT. (**b**) Exemplary DNA-PAINT image. (**c**) Corresponding histogram of intermark distances. (**d**) Mean distances with standard deviations of different DNA-PAINT images using different regions of the same sample at different time points over several days showing reproducibility and precision. (**e**) Ishikawa-diagram of potential measurement uncertainty contributions acting on the resulting distance. Black printed influences are considered later in more detail in the course of this manuscript while red printed influences turned out to be negligible for this work. A detailed discussion on the single individual influences can be found in Table S5 of the Supplemental Information.

Since then DNA origami nanorulers have been used to demonstrate the abilities of new superresolution microscopes by microscope manufacturers and they spread into superresolution labs worldwide. Researchers use them as positive controls for training, to distinguish between sample induced measurement problems and setup related problems as well as to demonstrate the resolution of their setups and techniques. In an increasing number of publications, DNA nanorulers are used to demonstrate new modalities and techniques^14,18–33^ including results with the so far highest reported optical resolutions reaching molecular scales^15,18,19^. Figure 1a illustrates a common DNA origami design used for the superresolution technique DNA PAINT^33^ (DNA-based point accumulation for imaging in nanoscale topography^34^) with two fluorescent marks at predefined distances. DNA PAINT is based on the successive localization of single molecules. The necessary ON/OFF switching is achieved by transient binding of short fluorescence labelled oligonucleotides (6–11 nucleotides) to the target structure that is equipped with complementary DNA strands. When a short DNA strand binds to the complementary DNA sequence on the target structure a fluorescence burst is observed. Localizing the center of the diffraction limited spot yields the position of the binding site with precision that is mainly dominated by the optical resolution and the number of detected photons during this binding event^33,35^. As the binding is thermally unstable, the DNA strand will diffuse back into solution after a period depending on the length of complementary DNA and the next binding event can be monitored to successively obtain all binding positions within diffraction limited areas of interest.

DNA PAINT is probably the most robust single-molecule localization technique as photobleaching does not play a role and the maximum photon budget can be extracted from the best dyes available yielding the highest resolution^15,19,33,36^. An exemplary superresolution image and a histogram of identified structures are additionally shown in Fig. 1b,c, respectively. The histogram of intermark distances in Fig. 1d shows that nanorulers yield almost identical results when measuring different regions of the same sample at different time points over several days. This proofs the precision that can be achieved with DNA origami structures but not the accuracy.

In this work, we go one step further and show how DNA origami nanorulers cannot only be test structures and positive controls but that they can be reference measurement standards as suggested recently^10,16,17^. This, however, requires a rigorous metrological analysis of the measurement process and results^10,16,17^. Metrology is the science of measurement and its application, building on the core concept of metrological traceability. To establish ubiquitously available reference samples such as the DNA nanorulers as traceable measurement standards, a documented unbroken chain of calibrations finally referring to SI units must be realised. This chain of calibrations includes a rigorous evaluation of all contributing measurement uncertainty contributions and a determination of the resulting measurement uncertainty for the measurand of interest. By using this metrological approach, the comparability of measurement results is achieved independent of when and where the measurement was carried out. The uncertainty estimation should preferably be done by following the *guide to the expression of uncertainty in measurement* (GUM)^37^ as it is common at metrology institutes and accredited labs.

It is well known that measurement uncertainties are quantified insufficiently by the standard deviation of a measurement which only accounts for stochastic uncertainty contributions such as noise. Usually, there are additional non-stochastic uncertainty contributions e.g. arising from the uncertainty of measurement parameters influencing the measurement result or the specified uncertainty of a calibrated measurement standard. In addition, the uncertainty of correction of systematic errors must be included in the uncertainty budget. Possible sources of uncertainty for superresolution measurements of DNA origami nanorulers are presented in an Ishikawa diagram in Fig. 1e and are sorted with respect to microscope uncertainties, sample uncertainties and uncertainties of the data analysis process. The Ishikawa diagram is the result of a brainstorming process and the uncertainty factors are further reduced to the most relevant ones. The relevant factors are shown in black and are further discussed in the course of the manuscript. An overview of the evaluation process is given in Table S5 of the Supplemental Information.

We developed a suitable traceability chain for determining the length of DNA origami nanorulers. For this purpose, the different uncertainty contributions shown in Fig. 1e have been studied and an uncertainty budget including stochastic and systematic uncertainties has been established. Finally, we present an exemplary uncertainty budget for the mean distance of an ensemble of nanorulers when measured with a typical superresolution microscope. Detailed information on this uncertainty analysis can be found in section 4 and in the sections 1–5 of the Supplemental Information.

Traceability is obtained using a commercially available stage micrometre that has been calibrated by the PTB^38^. The stage micrometre then serves to calibrate the pixel sizes in the object space of the microscope setup. Next, we calibrated the distance between marks using two exemplary nanoruler samples on three different setups. Summarizing, we enable the usage of ensembles of nanorulers as traceable distance measurement standards on the nanoscale for fluorescence microscopes with an expanded uncertainty (coverage factor *k* = 2, meaning a 95% coverage interval) of ± 2.4 nm following the GUM. Interestingly, we only used equipment from a regular SR-lab to demonstrate that such a metrological procedure can be introduced cost efficiently and without special technical requirements. Finally, DNA origami nanorulers are applied to probe offsets in multicolor imaging and to demonstrate that superresolution microscopes can achieve remarkable drift stability over several hours.

## From test-samples to nanoscale measurement standards

We introduce a calibration procedure to use DNA origami nanorulers as fluorescent nanoscale measurement standards beyond the diffraction limit. It is the most meaningful approach to perform this calibration with a superresolution microscope because other techniques such as atomic force microscopy could lead to results of poor comparability due to the requirement of adapted immobilization strategies and further difficulties when transferring the results from one measurement technique to the other. The most crucial aspect is that different chemical surroundings might be required for different analytical and imaging techniques. This can influence the measured distances^17^.

**Figure 2:**
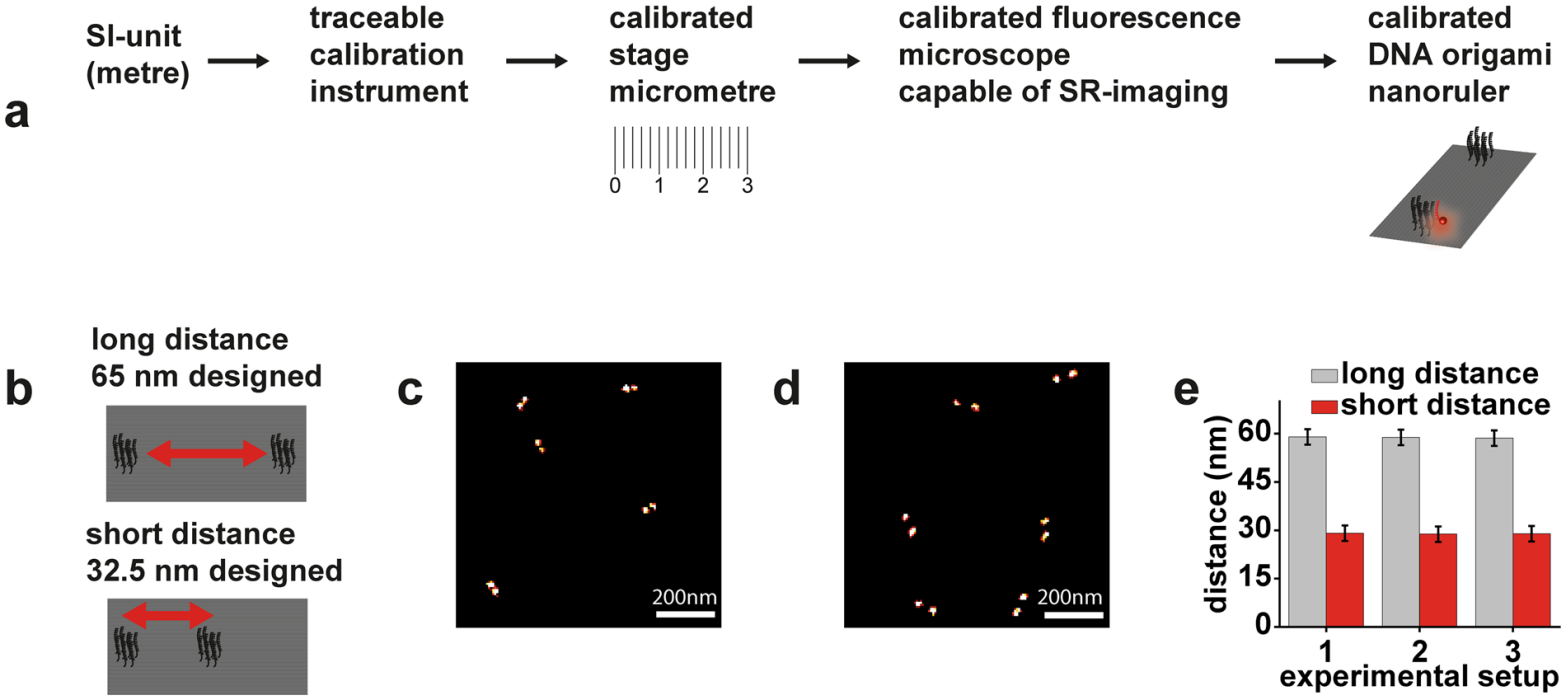
Distance calibration of DNA origami nanoruler samples. (**a**) Calibration chain starting from the SI-units (**b**) DNA-PAINT nanorulers with two different distances of 65 nm and 32.5 nm estimated by the nominal design of a rectangular DNA origami. (**c**) DNA-PAINT image of the short-distance nanorulers. (**d**) DNA-PAINT image of the long-distance nanorulers. (**e**) Calibration results of the two different samples using three different experimental setups. Error bars are expanded distance uncertainties calculated by following the GUM.

In this work, we use three objective-type total internal reflection fluorescence (TIRF) microscopes suitable for localization based SR microscopy. The length scale to determine the distances is given by the pixels of the EMCCD-camera used as a detector in the object space, i. e. the physical pixel pitch of the camera in the image space times the position dependent transversal demagnification of the imaging system. The actual pixel size in object space is determined by means of a calibrated stage micrometer. A scheme of the traceability chain to the SI units of the pixel sizes is depicted in Fig. 2a. A detailed description of the pixel size calibration can be found in the Supplemental Information, here we summarize the outcome.

The first step was the calibration of defined intervals of a commercially available stage micrometre (Präzisionsoptik Gera) that has a nominal pitch of 10 µm. A number of defined intervals of this structure were calibrated by the PTB Braunschweig with a traceable UV-microscope^38,39^, which offers an expanded uncertainty (coverage factor *k* = 2) of down to 6 nm for pitch measurements. However, due to the limited line edge quality of the used stage micrometre the calibration of individual 10 µm pitches yielded an expanded uncertainty (coverage factor *k* = 2) of 30 nm.

The calibrated pitch structures have been measured with the widefield microscopes to calibrate the pixel size of the EMCCD cameras. As shown in the Supplemental Information the expanded uncertainty of the mean pixel size is about 1%. The dominating uncertainty contribution is due to distortions of the microscope optics: the pixel size depends on the local position in the microscope’s field of view. In principle, this position dependent pixel size could be corrected, but in this work we omit this option. Instead, we consider the pixel size variation as an uncertainty contribution. Another major uncertainty contribution is due to the rather simple stage micrometre with weakly defined edges. Hence, a significant improvement of the pixel size calibration would be possible with more sophisticated calibration standards and methods. However, an important motivation of this work is to enable a calibration process that is easy to use for every superresolution lab and does not require a specialized metrology lab. The stage micrometre chosen is a cost-efficient solution that satisfies the requirements and the abstinence of correcting the position dependent magnification disables the need for an expensive scanning stage and an extra calibration step. Furthermore, it is our goal to unravel the uncertainty budget using a typical SMLM-setup to identify aspects that should be improved for advanced metrological applications.

As nanorulers, we chose DNA-PAINT nanorulers based on a rectangular DNA origami with two marks consisting of nine single-stranded DNA protrusions serving as DNA-PAINT binding sites. For a rough design with approximated intermark distances, the centre-to-centre distance between marks was estimated assuming a distance of 0.34 nm between the nucleotides along the DNA double helix and 3 nm between the centres of adjacent helices. We employed nanorulers with an approximated distance of 65 nm between the two marks (already used for the data displayed in Fig. 1) and of 32.5 nm. Sketches of the designs are shown in Fig. 2b.

The two nanoruler samples were measured (see Fig. 2c,d) with one of the calibrated TIRF microscopes. The measured mean intermark distances were 59.0 nm for the long nanoruler and 29.1 nm for the short nanoruler as depicted in Fig. 2e. These values are considerably smaller than expected from the design data, but one has to note that the microscope measures the 2D projection of the nanoruler which would result in smaller values in case of bending of the origami or due to deviations from a perfect horizontal orientation (tilt). In addition, the length of the intermark distances is influenced by environmental influences, e.g. salt concentration^17^. The error bars in Fig. 2e indicate the expanded measurement uncertainty (*k* = 2), calculated according to the GUM. It is 2.4 nm for each measurement as will be presented in detail in the next chapter.

To show that the DNA origami nanorulers can also serve as ubiquitous standards, we measured the same samples on the other two calibrated TIRF setups and found 58.8 nm and 58.6 nm for the long distance, and 28.8 nm and 28.9 nm for the short distance as also shown in Fig. 2e. The deviations are of the order of ~1 nm and clearly within the uncertainty associated with the measurement.

### Estimation of measurement uncertainty of nanoruler distance

To estimate the uncertainty of the nanoruler distance measurement, in principle all potential input quantities as shown in Fig. 1e must be considered and discussed. According to the GUM, a model of the measurement has to be set up which describes the functional relationship between the measurand and the input quantities on which the measurand depends. This functional relationship also contains all corrections or correction factors that can contribute significantly to the uncertainty of the measurement result.

First step is to define the measurand (here: the distance one is interested in). The length of a nanoruler with two marks is given as the distance between the centres of these marks. With the microscope setups used here, the 2D-projection of this distance (denoted as ${a}_{{proj}}$) is measured which is shorter due to bending of the nanoruler or tilting of the nanoruler with respect to the glass surface. The measurement of the projected distance of a single nanoruler can be described in a general form as

1 $${a}_{proj}=P(d+\sum _{j}\delta {d}_{j})$$

here $P$ is the pixel size (unit: nm/pixel), $d$ the measured distance (unit: pixels) and ${\sum }_{j}\delta {d}_{j}$ is the sum of different corrections (all in pixel units) which will be discussed below in more detail. On the supposition that all input quantities are independent, the formal uncertainty evaluation according to the GUM yields:

2 $${u}^{2}({a}_{proj})={u}^{2}(P)\cdot {(d+\sum _{j}\delta {d}_{j})}^{2}+{P}^{2}\cdot ({u}^{2}(d)+\sum _{j}{u}^{2}(\delta {d}_{j}))$$

The uncertainty $u(P)$ comes from the pixel-size calibration, while the other uncertainty contributions are caused by various influencing experimental parameters and the data evaluation. Eventually, we are interested in the mean intermark distance of an ensemble of nanorulers, i.e. $\bar{{a}_{proj}}$ and the uncertainty associated with this mean distance, i.e. $u(\bar{{a}_{proj}})$. An important issue is to rationalize if sample related influences on the measurement cause random or systematic uncertainties. The effect of random influences will be averaged out when the mean distance of a large number of nanoruler distances is determined. Systematic effects, however, act on each nanoruler measurement in the same or very similar manner, examples are the errors of the measurement of the pixel size or environmental influences.

Equation (3) shows the more detailed model equation used for our uncertainty analysis of the mean distance of an nanoruler-ensemble. Here, we concentrate on a few input quantities which – for our knowledge at the time being - are thought to contribute significantly to the uncertainty budget of the mean nanoruler distance. Equation (3) considers the different implications of random and systematic effects on uncertainty of the mean distance.

3 $$\overline{{a}_{proj}}=P(\frac{1}{n}\sum _{k}({d}_{k}+\delta {d}_{fit,k})+\delta {d}_{model}+\delta {d}_{blink}+\delta {d}_{env}+{d}_{fp})$$

In Equation (3), ${d}_{k}$ is the intermark distance of the the k-th nanoruler, and ${\delta d}_{{fit},k}$ describes a correction of this distance due to fitting errors caused e.g. by noise in the image data. The expectation value of this correction can be assumed to be zero, but it has an uncertainty which is directly obtained from the fitting routine output data. The term ${\delta d}_{{model}}$ corrects for residual imperfectness of the fit model. Again, we assume here that the expectation value of this correction is zero, but assign an uncertainty to it. Deviations of the intermark distances due to limitations of the blinking kinetics (i.e. if two molecules within a diffraction limited area are simultaneously “ON” and identified as a single “ON” event by the algorithm) are represented by ${\delta d}_{{blink}}$ and disturbances due to environmental influences are taken into account by ${\delta d}_{{env}}$. The term ${\delta d}_{{fp}}$ corrects the influence of non-excludable artefacts coming from false-positive/impurity signals that are misinterpreted as nanoruler signals. The expectation value of this correction is assumed to be zero, but for the uncertainty estimation we need to assign an appropriate uncertainty to this input quantity. In the following paragraphs – and in more detail in the Supplemental Information – we discuss these uncertainty contributions.

It is a helpful fact that superresolution localization can be simulated properly with Monte-Carlo methods to get a theoretical approximation of the system. This enables the variation of only one parameter while keeping all other parameters constant so that the influence of different effects can be rationalized. To this end, we customized a LabView based Monte Carlo simulation program to investigate effects such as photon numbers, labeling efficiency, localization numbers and blinking kinetics whether they are only influencing the statistical noise of ${d}_{k}$. The simulations show that the influence of the blinking kinetics and especially the ratio of t^on^/t^off^ does not average out when measuring many nanoruler distances. A high t^on^/t^off^ value goes along with increased probability that two molecules in a diffraction limited area are simultaneously emitting. As the blinking kinetics is well-controlled in DNA-PAINT, corresponding systematic shortening of the distances can, in principle, be reduced to an almost negligible amount (see Figure S4d in the Supplemental Information). Still, we considered the random uncertainty of our kinetic simulations of about 0.1 nm for realistic conditions: within this small interval we cannot resolve weather two molecules simultaneously bound are an uncertainty contribution of the mean intermark distance or not.

The influence of salt concentration on the nanoruler distance has been shown recently^17^. It depends, amongst others, on the geometry of the DNA origami. However, it has also been shown that distances are reproducibly obtained for a defined salt concentration, hence it is set as a fixed calibration parameter. The same holds for the temperature: small temperature gradients and changes of other environmental parameters result in sample drift that increases the uncertainty of spot centers. In our experiments, corresponding changes of the nanoruler distances are reduced to a level of below 0.5 nm (which is the detection limit for sample drift related influences on our setup) by drift suppression, comparable to previous work^15,17,31^. Hence, the corresponding uncertainty contribution ${u(\delta d}_{{env}})$ is not larger than 0.5 nm.

A more significant systematic uncertainty contribution ${u(\delta d}_{{fp}})$ comes from the analysis process and is based on the limitation that nanorulers are identified by signals that are likely to be nanorulers. At proper data quality the probability that such a signal comes from a nanoruler is near 100%, but in practice this value is not reached. The effect of the uncertainty contribution ${u(\delta d}_{{fp}})$ on the mean nanoruler distance is estimated to be about 0.9 nm by comparing software-automated and manual structure picking as shown in Figure S6. While both methods deliver reasonable results, automatic software filters cannot be perfect and pick a small subset of false structures while manual picking can be subjective. Furthermore, also manual picking cannot exclude improbable, but not excludable false positive signals that artificially look like nanorulers.

Another important aspect is the uncertainty coming from the algorithm used to calculate the single distances ${a}_{{proj}}$. In this work, we used the modification of a recent model^10,17^ (see Figure S7 and accompanying discussions) that shows improved fit quality for smaller distances. Based on the model there is a random uncertainty ${u(\delta d}_{{fit},k})$ of the single fit parameters and a systematic uncertainty ${u(\delta d}_{{model}})$ of the algorithm itself, representing its imperfect description of the data. A detailed discussion of the individual uncertainty contributions can be found in the Supplemental Information that is summarized in Table S5.

An interesting opportunity of the simulations is estimating the impact of a number of random parameter variations because these effects are hard to determine experimentally. This can be done by varying experimental parameters around their typical values and by comparing the resulting standard deviation (SD) obtained from the simulated distance statistics with the experimental SD. The simulated data yield a significantly smaller SD of about 1 nm compared to the experiment that typically yields a SD of 4 to 5 nm for the nanorulers used for this calibration process. This means that there are further random uncertainty contributions. Explanations could be distance variations between the single nanorulers (inhomogeneous broadening) or varying camera pixel sizes or varying pixel sensitivities. To test for the possible role of inhomogenous broadening, we performed additional simulations with a Gaussian distributed input distance instead of a fixed one. The results can be seen in Figure S5, showing that a realistic standard deviation can be obtained in this way. Consequently, we assume that the main contribution to the random uncertainty are possibly small structural/orientational variations of the nanorulers.

Based on this analysis we set up an example measurement uncertainty budget for the measurement of the mean distance $\bar{{a}_{proj}}$ of a nanoruler with a nominal distance of 60 nm and assuming a measurement of n = 1000 nanorulers. The uncertainty ${u(d}_{k})$ was set to 0.05 pixel (corresponding to 5 nm in nanoruler distance), and the uncertainty ${u(\delta d}_{{fit},k})$ to 0.0035 pixel (corresponding to 0.35 nm in nanoruler distance). The result is shown in Table 1.

**Table 1:** Example measurement uncertainty budget for the measurement of the mean nanoruler distance using setup no. 1.

Input quantity	Standard uncertainty of input quantity	Uncertainty contribution to ${\boldsymbol{u}}(\bar{{{\boldsymbol{a}}}_{{\boldsymbol{p}}{\boldsymbol{r}}{\boldsymbol{o}}{\boldsymbol{j}}}})$ in nm
$P$	0.5 nm/pixel	0.3
${d}_{k}$	0.05 pixel	0.16
${\delta d}_{{fit},k}$	0.0035 pixel	0.01
${\delta d}_{{env}}$	0.005 pixel	0.5
${\delta d}_{{blink}}$	0.001 pixel	0.1
${\delta d}_{{fp}}$	0.009 pixel	0.9
${\delta d}_{{model}}$	0.005 pixel	0.5
Expanded measurement uncertainty (*k* = 2): *U* = 2.4 nm		

### Benchmarking color channel match and drift stability with DNA origami nanorulers

Besides the use as nanoscale distance standard, we present two examples how one can further use DNA origami nanorulers to gain quantitative information about the performance of a microscope. First, we use the nanorulers to probe the correction of mismatches between color channels in multicolour imaging. Next we use them to show that a TIRF-microscope used for single-molecule localization microscopy is capable of 6 hours imaging without drift correction while keeping a resolution of better than 20 nm.

**Figure 3:**
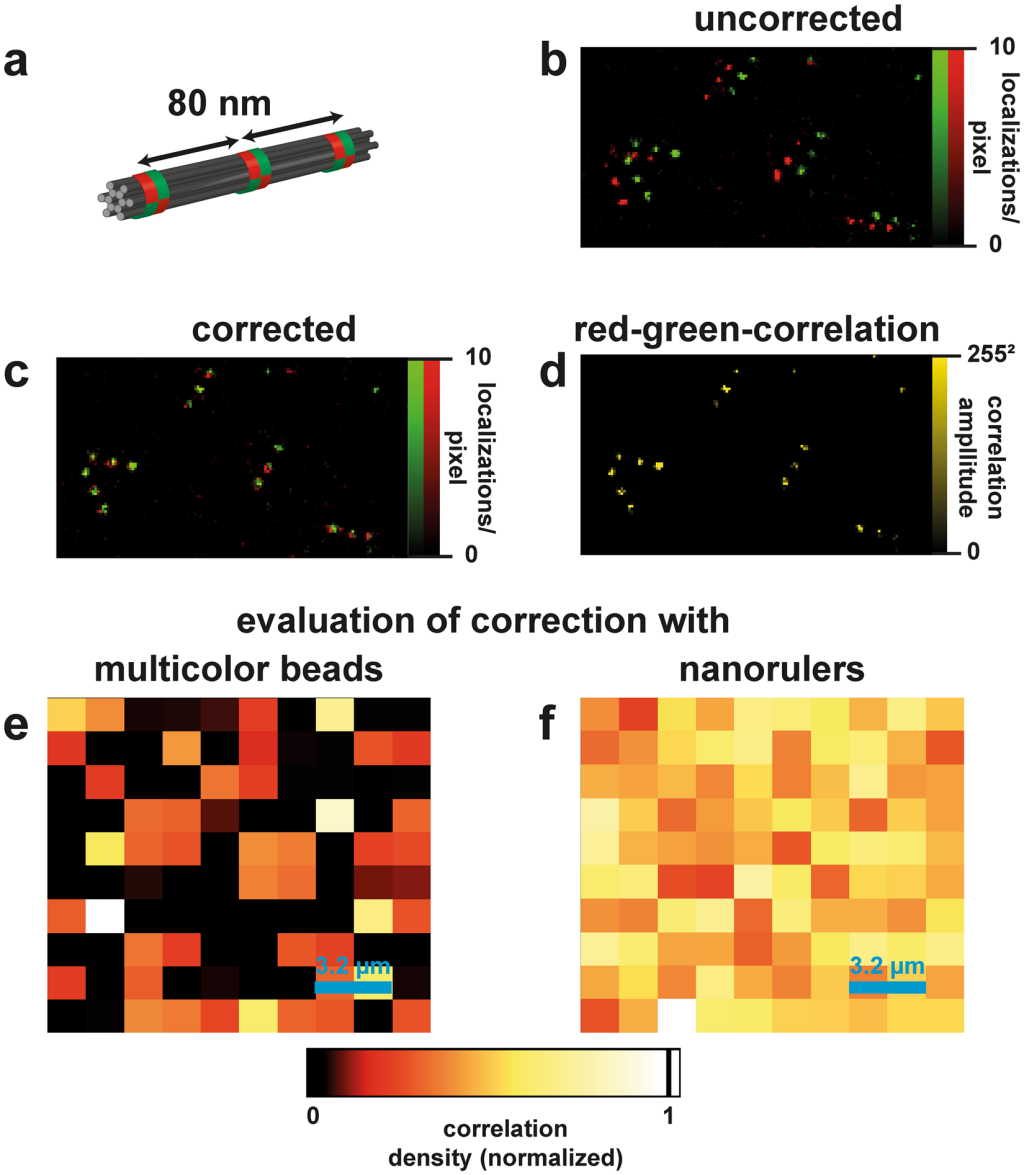
Correction of chromatic shift and evaluation with nanorulers. (**a**) Sketch of a GATTA-PAINT 80RG nanoruler used for this experiment. (**b**) Uncorrected merge of red and green SR-images showing a clear shift of ~90 nm. (**c**) Corrected overlay of the images. (**d**) Correlation image obtained by multiplying the red-and green values of the corrected image. Merged structures give a value > 0. (**e,f**) Full-size correlation images of multicolor beads (**e**) and nanorulers (**f**) showing the correlation density (summed up correlation amplitudes within 1.6 × 1.6 µm²-squares, normalized to maximum) in order to evaluate the correction quantitatively. Multicolor beads are limited to low surface densities while nanorulers show the desired homogenous correlation over the field of view within statistical noise.

It is generally known from multicolour superresolution imaging that the chromatic aberration correction might not hold anymore for sub-diffraction limited distances and needs calibration measurements in order to correct the differences between different colour channels. Besides aberration, a shift of the colour channels with respect to each other is often observed as a result of wedge effects of the optical components in the detection path. The result can be probed with multicolour DNA origami nanorulers (here commercially available GATTA-PAINT 80RG nanorulers, GATTAquant GmbH, depicted in Fig. 3a). Exemplary superresolution DNA PAINT images with substantial shift between the colour channels are shown in Fig. 3b. The shift has to be corrected and the correction is evaluated when accurate quantitative results are required. To this end, a superresolution image of multicolour-beads (in our case GATTAbeads RG, GATTAquant GmbH) distributed over the field of view was acquired. The centre coordinates of the corresponding signals were then used for correction.

Here we exemplary assume that the images can be corrected by a constant vector (shift) and a linear dependence on the field coordinates x and y representing a potential effect of chromatic aberrations. For correcting for the mismatch, the green coordinates were transformed by the equations 4 and 5. As modelling of the exact influence of the optical components on the mismatch would be demanding we chose this simple approximation and empirically tested the model with DNA origami nanorulers. This provides a simple validation of the model to enable properly matching color channels on the few-nanometer scale.

4 $${x}_{red}(x,y)=a\cdot {x}_{green}+b\cdot {y}_{green}+c$$

5 $${y}_{red}(x,y)=d\cdot {x}_{green}+e\cdot {y}_{green}+f$$

With the bead data, the constants a, b, c, d, e, and f were determined by analytically solving this equation system as shown in the SI. This yields a, e ≈ 1 and b, d ≈ 0 in our case. This means that the major component of the shift is a constant vector that was subtracted from the coordinates of one colour channel. Again, the result is only valid for this setup and the analysis has to be performed for each setup separately. For our case, the correction is in accordance with a shift of the color channels with respect to each other as frequently encountered by imperfections especially of dichroic mirrors. An exemplary corrected image is shown in Fig. 3c. In order to evaluate the correction quantitatively we multiplied the red and green colour channel (RGB values) of a 10 nm-binned image in order to get a correlation image: in regions with no correlation, the value turns to 0 while it gets larger for correlated regions. It is important to ensure that the correction holds constantly for the full field of view. To probe this, we binned the correlation image in 1.6 × 1.6 µm² sub-regions and defined the correlation density: this is the sum of correlation amplitudes within these areas. In a proper correction it is expected to be constant within statistical noise, while an unsatisfying result would give a gradient over the field of view.

This analysis would in principle also be possible with multicolour beads but they have a big disadvantage here: due to their constant signal they have to be used highly diluted for surface densities that allow individual localization. Thus, they do not give a proper density of data points for this analysis. DNA origami nanorulers are blinking instead so they can be imaged at much higher surface densities by superresolution. The corresponding results can be seen in Fig. 3e and f: while the multicolour beads do not allow to make a quantitative analysis of the correlation density over the field of view, the nanorulers can properly demonstrate that it is constant within statistical noise. We therefore suggest the nanorulers as a test structure to evaluate corrections like this.

**Figure 4:**
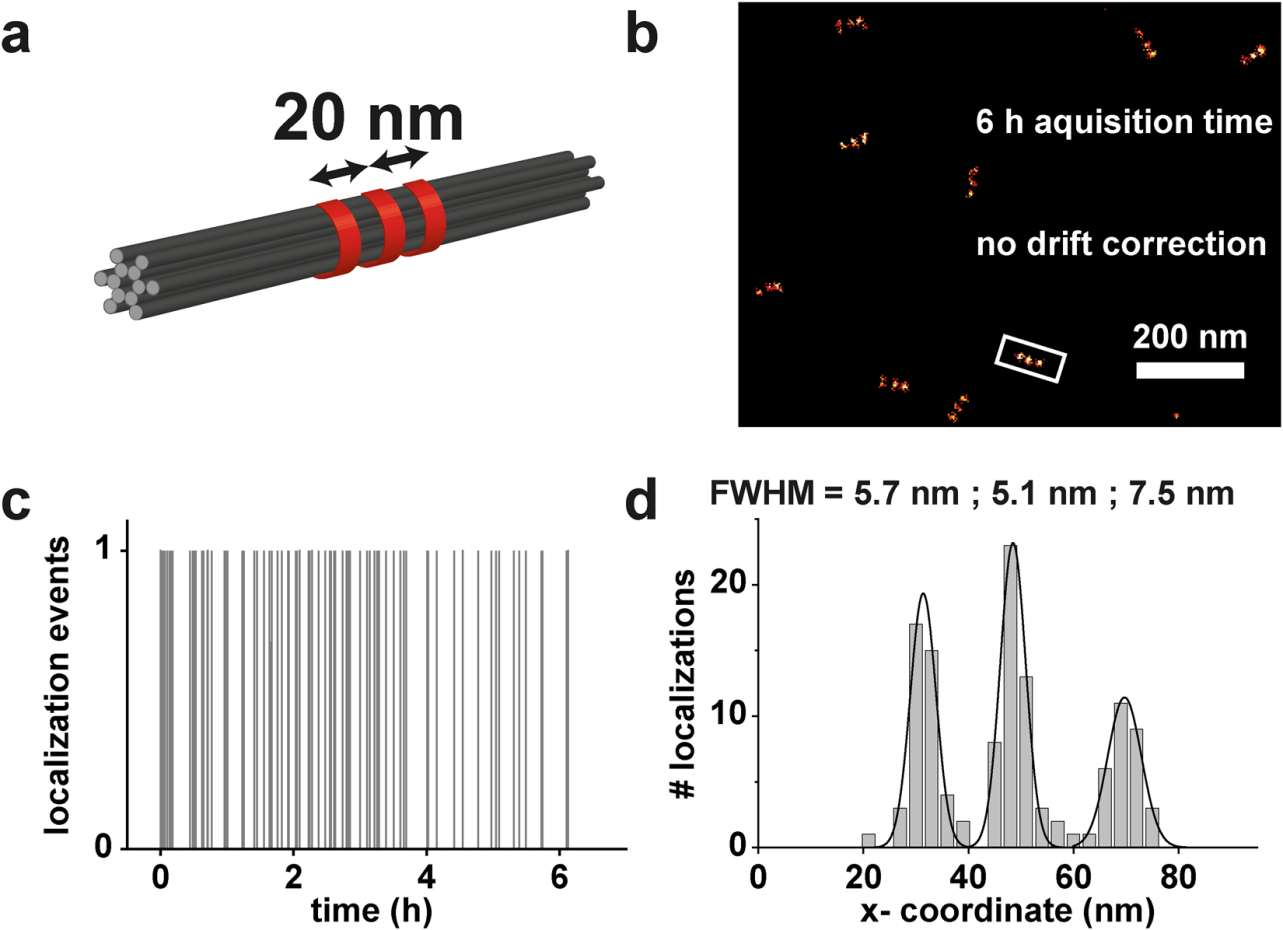
Evaluation of drift stability and resolution over time using nanorulers. (**a**) Sketch of a GATTA-PAINT HiRes 20 R nanoruler used for this experiment. (**b**) DNA-PAINT image of the nanorulers was acquired over a total time of 6 hours without the need of drift correction. (**c**) Localization transient of the white framed nanoruler in (**b**) indicating that the structure has been localized over the whole acquisition time without a detectable influence of drift. (**d**) Crossection of the white framed nanoruler in (**b**) including analysis of the spot widths by Gaussian fits.

Besides the accuracy of the optics another important parameter for the performance of a superresolution microscope is its mechanical stability. For long measurement times, the focus might not be stable and xy-drift can occur. An autofocus system, enabled by a piezo-controlled objective stage, and a feedback loop can stabilize the focus. The resulting movements are causing sample drift that has to be corrected, e.g. by tracking fiducial markers. An exemplary correction for GATTA-PAINT HiRes 20 R nanorulers can be found in the SI. Passively, focus and drift problems can be suppressed by reducing the mechanical path length of the focussing system. Practical solutions are commercially available (see setup description in the methods part). Ideally, these mechanical stabilizers are used in combination with a stabilized optical table to suppress vibrations in a temperature stabilized environment. The performance of passively stabilized microscopes was benchmarked by DNA origami nanorulers as illustrated in Fig. 4 using the GATTA-PAINT HiRes 20 R (Fig. 4a). The data shown demonstrate the drift stability (in our example over 6 hours) and additionally indicate the resolving power that is maintained during the measurement time interval (Fig. 4b–d). In our example we can show that a resolution of better than 20 nm is obtained for a 6 hour-DNA-PAINT measurement without the need of drift correction.

## Conclusion

In this work, we presented how DNA origami nanorulers can be calibrated and how they can be used for metrological applications. While the primary applications of DNA origami nanorulers are positive controls, microscopy demonstrations and training, here we show that their quality, reproducibility and robustness even enables traceability to the SI units. Thus, DNA nanostructures have the potential to become prime examples of self-assembled reference structures and traceable ubiquituous standards for length measurements and beyond. A direct practical application is the quantitative determination of the resolution of SR-microscopes and methods. To this end, we developed a procedure how to calculate a combined measurement uncertainty using the GUM when imaging DNA origami nanorulers by single-molecule localization DNA-PAINT. However, it should be kept in mind, that only the projections of the DNA nanoruler pitches in the xy-plane perpendicular to the optical axes of the microscope are calibrated. Therefore, the applicability and transfer is essentially dependent on the adequate preparation (alignment) and the sufficient flatness of the DNA origami. In the future, extension for quantitative 3D measurements is envisioned^16^.

Our calibration approach can be transferred to other superresolution microscopy systems such as stimulated emission depletion (STED) or structured illumination microscopy (SIM) when methodical differences are taken into account by adjustments in details of the calibration procedure. Every aspect has to be treated carefully when the measurement uncertainty budget is set up in order to perform a proper calibration. Furthermore, this uncertainty analysis can serve as an example how to apply existing procedures and calibration standards of metrology institutes to standardize novel measurement methods and samples and thereby improving comparability. In a more specialized point of view this work is another important step to standardize the use of DNA origami nanorulers to make them an easy-to-use tool for interdisciplinary researchers using fluorescence microscopy. To demonstrate this character of the samples we additionally used the nanorulers for comparing the matching of different colour channels or the mechanical stability of a fluorescence microscope capable of superresolution microscopy by successive single-molecule localizations.

## Methods

### Sample preparation

Gatta-PAINT 80RG, GATTA-PAINT HiRes 20 R nanorulers, and GATTAbeads RG were provided by GATTAquant GmbH Braunschweig, Germany.

Rectangular DNA-origami structures (d_designed_ = 65 nm and 32.5 nm) were synthesized by mixing p7249 scaffold (17.6 nM) with unmodified and modified DNA staple strands in folding buffer (5 mM Tris, 1 mM EDTA, 12.5 mM MgCl_2_, pH 8) to a final volume of 100 µL. The molar excess of unmodified and unmodified staple strands was 10 and 30, respectively. The folding process was carried out under the following thermal conditions: 90 °C for 15 min, 89–20 °C, 1 min per °C. Folded DNA-origami samples were purified by agarose gel electrophoresis (1.5%, 80 V, 90 min). For all DNA-PAINT-measurements we used Nunc Lab-Tek chambered coverglasses (Thermo Scientific 155409, # 1.5). Prior to modification of Lab-Tek chamber with BSA-biotin (0.5 mg/mL in PBS, overnight at 4 °C), it was purified by 1 M KOH for 5 min. After three washing steps with PBS (137 mM NaCl, 2.7 mM KCl, 10 mM KH_2_PO_4_/K_2_HPO_4_) it was incubated with NeutrAvidin (0.5 mg/mL in PBS) for 1 h at room temperature. Purified DNA-origami sample was diluted 1:20 with PBS and incubated in Lab-Tek chamber for ca. 10 min. Afterwards, it was washed three times with PBS and additionally with PBS containing 10 mM MgCl_2_ as imaging buffer. In dependence of microscope the length of DNA-imager strand of 8 or 6 bases was used for superresolution imaging. For all measurements the imager strand was diluted to 5 nM with imaging buffer. The mean room temperature for all measurements at different microscopes was 22.7 ± 0.3 °C.

### Data Acquisition and image processing

All DNA-PAINT images have been acquired at an excitation intensity of ~4 kW/cm² and an EM-Gain of 5. The integration time and frame number have been 100 ms and 12000 frames for the measurements with rectangular nanorulers, 50 ms and 20000 frames for the Gatta-PAINT 80RG as well as 500 ms and 45000 frames for the GATTA-PAINT HiRes 20 R. Single-molecule signals have been localized with a self-customized MATLAB-software. Analysis of the nanoruler-SR-images has been performed with self-customized Labview-software and the Gattanalysis software tool (GATTAquant GmbH). Correction of chromatic aberration has been done with the GATTAquant-chroma-fix software.

### Experimental setups

Setup no.1 is a custom-built total internal reflection fluorescence (TIRF) microscope, based on an inverted microscope (IX71, Olympus) Excitation was carried out at 644 nm with a 150 mW laser (iBeam smart, Toptica Photonics) spectrally filtered with a clean-up filter (Brightline HC 650/13, Semrock). For two-colour imaging an additional 532 nm/1 W fibre laser (MPB Communications) also filtered with a clean-up filter (Z532/647 × , Chroma) was used. The laser beam is coupled into the microscope with a dual-colour-beamsplitter (Dual Line zt532/640 rpc, AHF Analysentechnik) and focused on the backfocal plane of an oil-immersion objective (100 × , NA = 1.4, UPlanSApo, Olympus) aligned for TIRF illumination. The fluorescence light is guided through an additional 1.6 × optical magnification lens, an emission filter (ET 700/75, Chroma for red excitation or BrightLine 582/75, AHF Analysentechnik for green excitation) and finally focused on an electron multiplying charge-coupled device (EMCCD) camera (Ixon X3 DU-897, Andor) for detection. Sample drift has been suppressed with an actively stabilized optical table (TS-300, JRS Scientific Instruments) and equipped with a nosepiece stage (IX2-NPS, Olympus). The calibrated pixel size is 101.05 ± 1.0 nm/pixel.

Setup no.2 is also a custom-built total internal reflection fluorescence (TIRF) microscope, similar to the first one. Deviating it has at 639 nm laser (iBeam smart, Toptica Photonics, 150 mW), a Brightline HC 642/10 clean-up filter, a 647 nm long-pass filter (Semrock Razor Edge) in the detection path and no actively stabilized optical table. Furthermore, it has a beamsplitter device (TripleSplit, Cairn) between the microscope and the EMCCD-camera that was however used in single-line mode here. It influences the calibrated pixel size to be 95.6 ± 1.2 nm/pixel.

Setup no.3 is a commercial Leica GSD system. For excitation of the fluorophores it has a fibre laser (MPB Communications, 642, P_max_ = 500 mW) fibre-coupled via a motorized objektive-TIRF module (Leica DMi8/AM TIRF MC) and a Leica 647 HP-T filter cube into an 160 × oil-objective (Leica HCX PL APO, NA = 1.43). The fluorescence light is detected by an Andor iXon Ultra 897 EMCCD-camera. Sample drift has been suppressed by a Leica SUMO (suppressed motion)-stage and table actively stabilized by piezo elements (Table Stable TS-140-LP). The calibrated pixel size is 99.9 ± 1.1 nm/pixel.

### Data availability statement

The datasets generated during and/or analysed during the current study are available from the corresponding author on reasonable request.

## Electronic supplementary material

Supplemental Information
