# Supplementary material for: Using DNA origami nanorulers as traceable distance measurement standards and nanoscopic benchmark structures
Source: Sci Rep. 2018 Jan 29;8:1780. doi: 10.1038/s41598-018-19905-x (PMC5789094; doi:10.1038/s41598-018-19905-x)
Supplement: Supplementary file 1 — Supplemental Information [file 41598_2018_19905_MOESM1_ESM.pdf]

Supplemental information

Using DNA origami nanorulers as traceable distance measurement standards and nanoscopic benchmark structures

Mario Raab[1,3], Ija Jusuk[1,3], Julia Molle[1], Egbert Buhr[2], Bernd Bodermann[2], Detlef Bergmann[2], Harald Bosse[2], Philip Tinnefeld[1,3]

[1]Institute for Physical & Theoretical Chemistry, and Braunschweig, Integrated Centre of Systems Biology (BRICS) and Laboratory for Emerging Nanometrology (LENA), Braunschweig University of Technology, Rebenring 56, 38106 Braunschweig, Germany.

[2] Physikalisch-Technische Bundesanstalt (PTB), Bundesallee 100, 38116 Braunschweig, Germany.

[3]Department of Chemistry and Center for NanoScience, Ludwig-Maximilians-Universitaet Muenchen, Butenandtstr. 5-13, 81377 Muenchen, Germany.

1. Pixel size calibration

1.1 Calibration of the stage micrometre

To calibrate DNA origami nanorulers first the EMCCD camera pixel size in the object space of the applied microscope system was traceably calibrated, because the camera pixels represent the length scale used for distance measurements in superresolution (SR) microscopy. A pixel size calibration is commonly done using a traceably calibrated stage micrometre.

The calibration of the stage micrometre was performed by the optical linewidth calibration tool of PTB. This system is based on a modified commercial microscope (Zeiss AXIOTRON) and validated both for linewidths and pitch calibrations. The sample is Koehler-illuminated in epi-configuration at a central wavelength of 365 nm (spectral bandwidths 20 nm) with an illumination numerical aperture of NA_C = 0.9 and imaged by a microscope objective with a lateral magnification of m = 150 and a numerical aperture of NA_O = 0.9. To realise a scanning microscope, a slit aperture is placed in the image plane in front of a photomultiplier. This aperture again is imaged de-magnified into the object plane by the microscope objective, where it has a size of 3700 nm × 90 nm. Linewidth and pitch measurement are based on the measurement of the transmitted light intensity using a photomultiplier, while the sample is moved highly precisely and interferometrically controlled in the focal plane using a one-dimensional high-precision nanopositioning stage. The edge positions are deduced from the measured signal profile using in case of pitch measurements a 50 % threshold criterion. The pitch of individual grating lines can be obtained as the difference of the left edge or right edge positions of the corresponding lines or as the mean value of both values.

1.1.1 The stage micrometre

For the pixel-size calibration we used a stage micrometre (Präzisionsoptik Gera, 2 mm in 200 parts (Figure S1a, b).

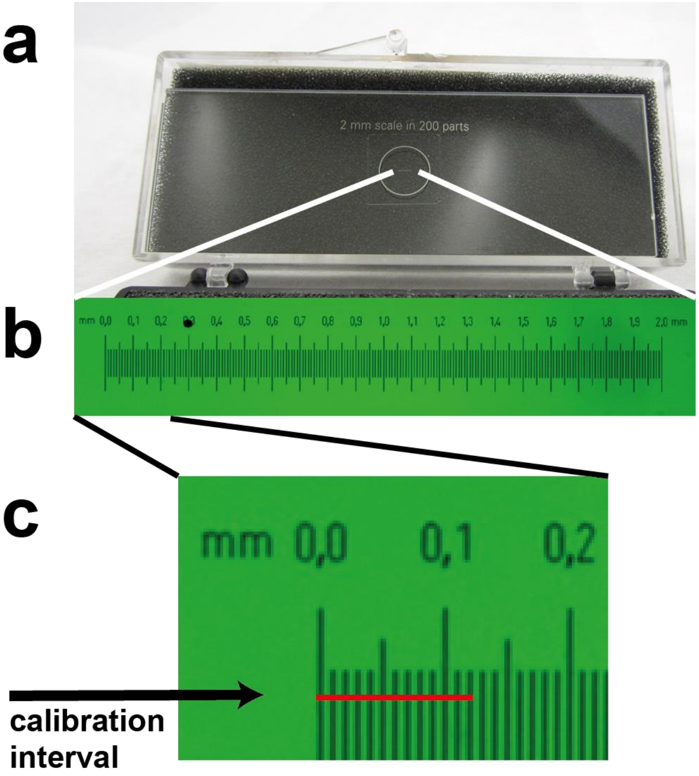

Figure S 1 a) Photo of the stage micrometre slide. b) Image of the scale acquired with transmitted light and 5x magnification. c) Interval on the scale used for pixel size calibration (red, 4 µm height, from 0.0 mm to 0.12 mm-line, 15 µm away from the edges).

This calibration sample is a cost-efficient and common tool for every microscopy lab. However, being handled in ordinary laboratory environment (and not in clean room facilities) typical pollution is identified within the area of the calibration structure. Furthermore, the lines show distinct structure errors at some places. These circumstances must be taken into account for the uncertainty evaluation of the calibration process.

1.1.2 Calibration Interval

The distances between the adjacent left edges and between adjacent right edges of the first 13 lines (line 0.0 mm to 0.12 mm) were calibrated. The middle of the scan diaphragm (length = 3.7 µm in object coordinates) is placed approximately 15 µm underneath the upper edge of the short pitch structures for measuring (Figure S1c). This measuring track is chosen such that no large structure errors occur within the track. Five measurement series each with 10 repetitions were carried out. Therefore, each value is the average of more than 50 single measurements. The measured distance values varied within 2 nm to 5 nm (standard deviation) depending on the line measured.

1.2 Pixel size calibration using the traceable stage micrometre

As the distances between the lines are known the calibration of the pixel size in the object space of the microscope system under test can be performed as sketched in Figure S2a. For this purpose, we placed the stage micrometre on the microscope stage with the lines of the scale aligned parallel to the pixel columns of the EMCCD-image and imaged it with transmitted white light. Then we analysed it within the calibrated interval using customized software based on LabVIEW 2011 to determine the pixel size and estimated the uncertainty following the GUM[1]. As shown in Figure S2b the distance between two lines of the stage micrometre is determined by measuring the difference d between the edge positions x_1 and x_2 of them.

$$d = x_1 - x_2$$

Due to diffraction and residual optical aberrations the edges are not infinitely sharp but show an intensity gradient (Figure S2c). Thus, an edge localization criterion is needed. For this purpose, we used the intensity contrast of the lines and the space between them. The mean intensities $\langle I_{space} \rangle$ as well as $\langle I_{line} \rangle$ were determined for both regions from pixel-columns sufficiently far away from the edge profiles (> 5 pixel) to ensure a negligible impact edge profiles. The intensity at the edge was then defined as the arithmetic average of these two mean values and the linearly interpolated pixel positions corresponding to this threshold I_τ were determined as illustrated in Figure S2d.

In this way, the distances d and the corresponding uncertainties were determined within a measuring window matching the size and position of the slit used in stage micrometre calibration (~4 µm / 40 pixels height, center 15 µm above the bottom of the lines). All values were averaged within this window. Every line-to-line distance was measured on 100 different images. The corresponding mean value $\langle d \rangle$ was used to calculate the pixel size P using the following model equation:

$$P = \frac{S_{ref}}{\langle d \rangle}$$

with

$$\langle d \rangle = d_{mean} - \delta d_{angle} + \delta d_{ER} + \delta d_{IP} + \delta d_{pos} + \delta d_{filter} + \delta d_{ab}$$

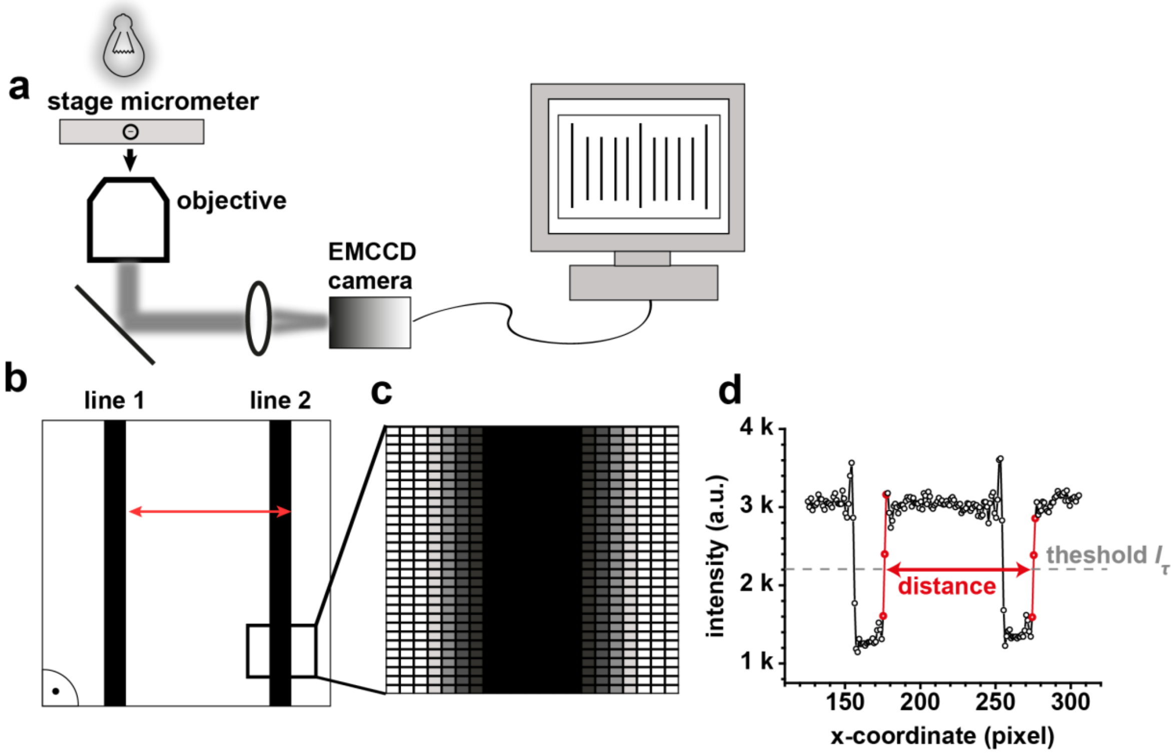

Figure S 2 a) The calibrated stage micrometre is placed on the objective of the microscope illuminated by transmitted white light. The EMCCD camera serves as detector. The edges are defined via their intensity. b) The distance is measured between corresponding edges. c) The edges show an approximately linear intensity gradient. d) To localize the edges the medium intensity on and between the lines is determined. The threshold (grey) was chosen to be the medium intensity between them. The pixel surpassing that threshold and the both surrounding pixels were identified (red) and fitted with a linear function. According to the fit, the position of the edge was then calculated as the corresponding coordinate to the threshold intensity.

The reference distance s_{ref} and its uncertainty is known from the stage micrometre calibration. In principle, the measured mean distance d_{mean} needs to be corrected for a number of effects caused by the non-perfect orthogonality of the lines to the pixel-rows (δd_{angle}), the roughness of the edges (δd_{ER}), impurities on the sample and optics (δd_{IP}), imperfect positioning of the measurement window (δd_{pos}), the used fluorescence filters (δd_{filter}) as well as by optical distortions or aberrations resulting in a position dependent lateral magnification (δd_{ab}). These aberrations can e.g. be the result of a non-perfect correction of field curvature as listed in Figure 1e. Apart from the effect of a non-perfect orthogonality, the specific numerical values of these corrections are not known exactly for a single measurement. It has been assumed that these corrections have the expectation value zero. Hence, for a specific measurement, the numerical value d_{mean} is unchanged. However, there are uncertainties assigned to these corrections which thus increase the uncertainty of the mean value $\langle d \rangle$.

For setup no.1 the pixel size P (here: P = 101.05 nm/pixel) and its uncertainty was determined based on a single line pair of the stage micrometre. The uncertainty budget is shown in Table S1. The expanded uncertainty using the coverage factor k = 2 is U(P) = 1 nm/pixel.

Table S 1 Measurement uncertainty budget for the mean pixel size P of setup no. 1.

Input quantity	Uncertainty of input quantity	Uncertainty contribution to pixel size P (nm/pixel)	Relative contribution (%)
s_{ref}	15.0 nm	0.15	9.67
d_{mean}	0.08 pixel	0.08	2.75
∂d_{angle}	$1.00 \cdot 10^{-3}$ pixel	0.001	0.00
δd_{IP}	0.05 pixel	0.05	1.07
δd_{ER}	0.03 pixel	0.03	0.39
δd_{pos}	0.02 pixel	0.02	0.17
δd_{filter}	0.2 pixel	0.20	17.19
δd_{ab}	0.4 pixel	0.40	68.76
Expanded measurement uncertainty (k = 2): U = 1.0 nm/pixel			

Looking at the relative uncertainty contributions it becomes obvious, that the position dependent magnification is the dominating uncertainty contribution here. Consequently, averaging over multiple pitches would not be effective here since it does not reduce this effect.

Table S2 summarizes the pixel size calibration results for all three setups used in this work.

Table S 2 Pixelsize calibration result for all 3 SR-setups

Setup no.	Pixel size (nm/pixel)
1	101.05 ± 1.0
2	95.6 ± 1.2
3	99.9 ± 1.1

2. Monte Carlo simulations of uncertainty contributions.

When it comes to distance measurements not only uncertainties due to the setup, but also uncertainties coming from the sample must be considered. These uncertainties can have a photophysical origin coming from the dye molecules or a structural origin coming from the DNA origami. Quantifying the single contributions is demanding because it means controlling all properties while probing a single one. Thus, an alternative approach is necessary to rationalize these influences.

To study the contributions of different measurement parameters on the distance measurement uncertainty we performed Monte Carlo Simulations, starting from the generation of signals, following the whole analysis path and terminating with the statistical analysis of multiple distance measurements on simulated nanoruler signals. A simplified scheme of the simulation algorithm is illustrated in Figure S3.

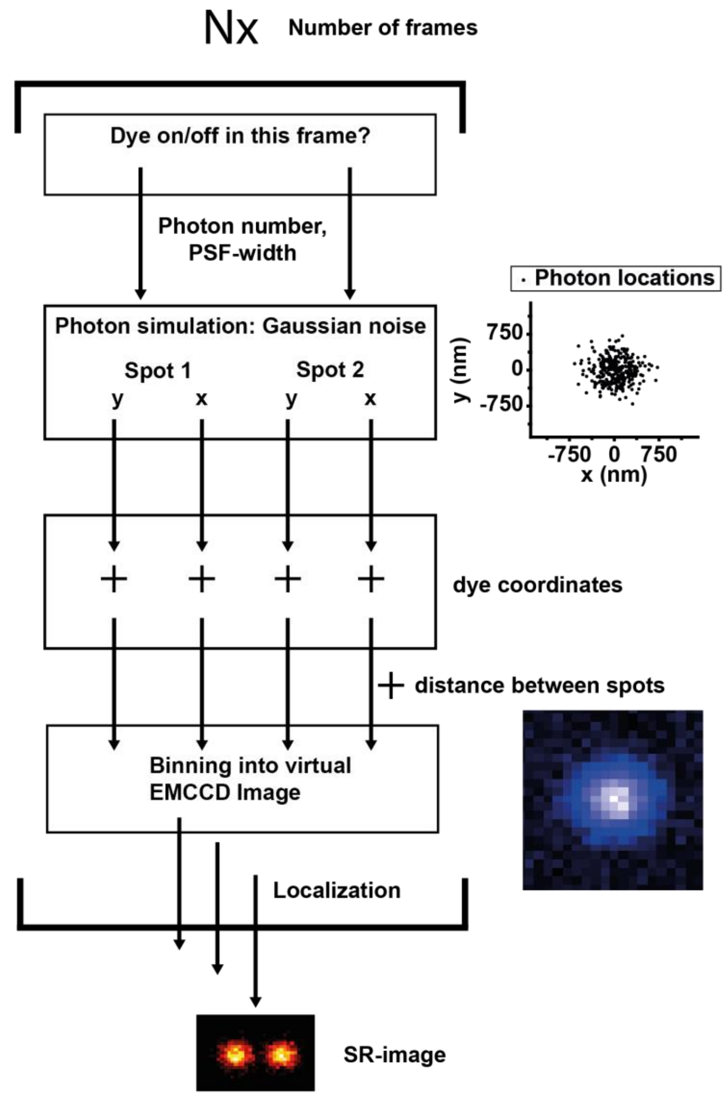

Figure S3 Programming scheme of the simulation tool.

The main advantage of the simulation is the ability to control every single parameter without changing others. We probed the influence of the labelling efficiency (Figure S4a) as structural influence. Furthermore, we probed the photon number (Figure S4b) and the localization per spot which can be interpreted as the influence of photobleaching or the acquisition time (Figure S4c) as photophysical influences. We also probed the influence of the blinking kinetics (namely the ratio of on- and off-time), see Figure S4d. The exact simulation parameters are listed in Table S3.

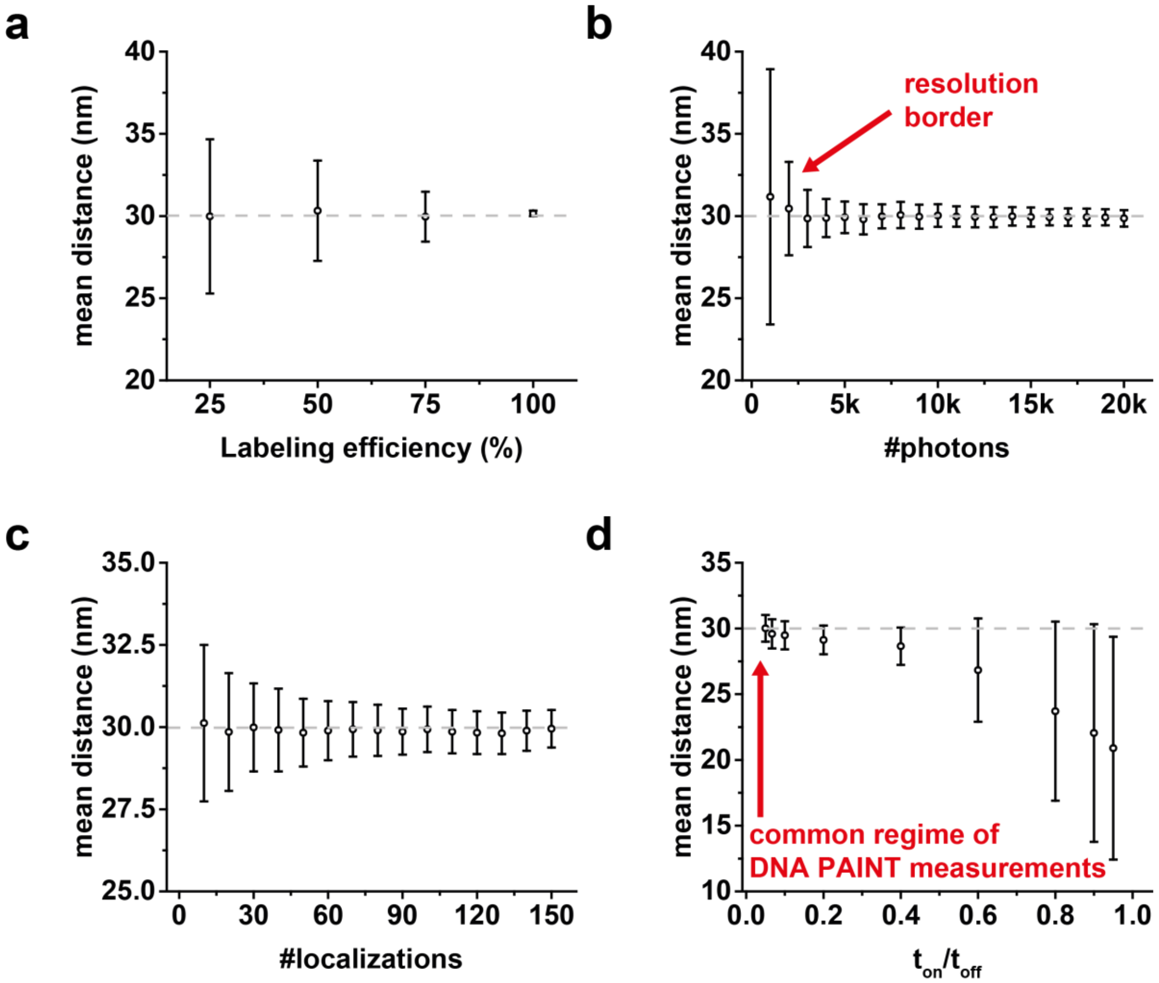

Figure S4 Simulated influence of labeling efficiency (a), photon numbers (b), localization count (c) and blinking kinetics (d). Dashed, grey lines indicate the designed distance (30nm). Error bars are standard deviations.

Table S3 Simulation conditions for the data shown in Figure S4. A "x" indicates that the simulation has been done for multiple values of the corresponding property (see graphs).

dependency	property	value
labelling efficiency	distance (nm)	30
	#photons	20000
	#frames/dye	50
	#dyes/mark	9
	labelling efficiency (%)	x
	t_{on}/t_{off}	no blinking limitation
#photons	distance (nm)	30
	#photons	x
	#frames/dye	50
	#dyes/mark	1
	labelling efficiency (%)	100
	t_{on}/t_{off}	no blinking limitation
#localizations	distance (nm)	30
	#photons	5000
	#frames/dye	x
	#dyes/mark	1
	labelling efficiency (%)	100
	t_{on}/t_{off}	no blinking limitation
blinking kinetics	distance (nm)	30
	#photons	5000
	#frames/dye	500
	#dyes/mark	1
	labelling efficiency (%)	100
	t_{on}/t_{off}	x

As mentioned in the manuscript the most important aspect is the question if the uncertainties are random or systematic. Random uncertainties are not problematic since their overall influence is experimentally observed and can be minimized by large statistics which is straightforward with DNA origami nanorulers. Systematic errors are much more critical. They must be corrected or suppressed by proper experimental conditions if possible. It is expected that all influences but the blinking kinetics produce random uncertainties because they blur the signals without shifting their centre. The simulations confirm these expectations with the following limitations:

The **photon number** can cause systematic distance uncertainties when it is so low, that the distance of the nanoruler is near the resolution border. For the simulated ruler length of 30 nm one should have at least ~3000 photons per camera frame. At our measurements conditions, we typically detected 18000 ± 3000 (mean±SD) photons per camera frame. Since this is far more intensity than needed to resolve a 30 nm distance the photon number is only contributing to the random uncertainty.

In case of the **labelling efficiency** it is assumed that every dye on the nanoruler has an equal missing possibility when the labelling efficiency is smaller than 100%. Unequal labelling efficiencies for the different strands could lead to systematic uncertainties.

It is generally known from localization microscopy that double localization can occur at improper **blinking kinetics**, giving a false localization between two emitters. This can lead to a systematic shortening of the measured distance. This is demonstrated by the simulation results in Figure S4d. Consequently, a low duty cycle is mandatory for achieving accurate distances. This influence can be neglected when t_{on}/t_{off} is tuned to be much smaller than 0.05. This is especially possible for DNA-PAINT because the method allows adjusting the blinking kinetics arbitrarily by choosing proper imager concentrations and binding sequences[2]. In our experiments, the on-off-ratio was about 0.02, i.e. in a regime where no significant shortening of the distances occurs. For our experiments, we however estimated an uncertainty $u(\delta d_{blink})$ representing a non-resolvable influence that could be caused by this effect.

The question arises whether these influences are the only ones acting on the measurement uncertainty or if there are other influences – most likely **geometrical inhomogeneities** increasing the uncertainty. To answer this question, we performed additional simulations with mixed influences. The values of the input parameters including their standard deviations were chosen to be in typical ranges known from experiments. They are listed in Table S4. Potential geometrical inhomogeneities were simulated by adding Gaussian distributed noise with defined standard deviations (SD) to the distance between the marks. The results for different SDs can be seen in Figure S5. While experimental SDs of the distance are in the order of ~4-5 nm simulations without distance-noise have an overall SD of ~1 nm. Output SDs comparable to the experimental results are only reached when also the SD of the input distance is chosen to be 4-5 nm.

Thus, it is likely that the main random uncertainty contribution in the experiments is geometrical inhomogeneity of the sample. The reason can be small defects of single structures as well as slight differences in their 3D orientation (note that we are just measuring the 2D-projection in this work). However, the reproducibility of the mean distances show that a statistical analysis of the structures is still reasonable for calibration purposes.

Table S4 **Simulation conditions for the data shown in Figure S5. The “x” indicates that the simulation was done for multiple values of the corresponding property (see graph).**

property	value	SD
distance (nm)	30	x
#photons	18000	3000
#localizations/dye	19	6
#dyes/mark	9	0
labeling efficiency (%)	77	28
t_{on}/t_{off}	0.02	0

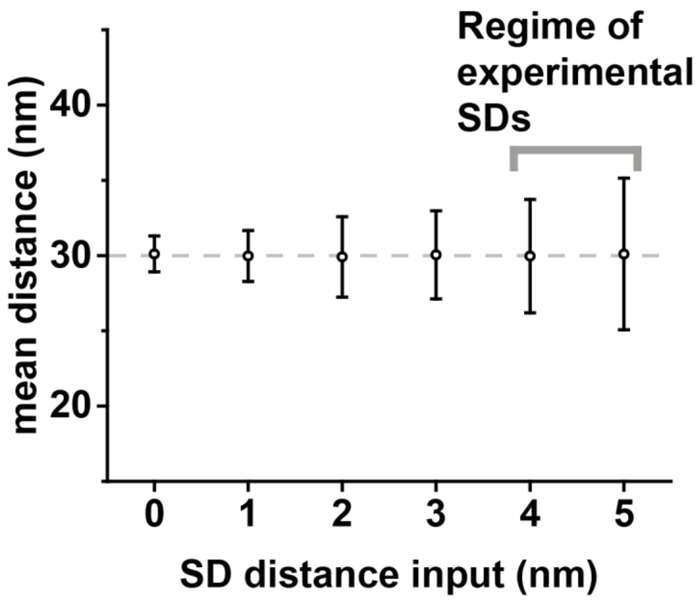

Figure S5 Simulation with experimental determined conditions at different noise levels of the input distance, representing geometrical deviations of single nanorulers. The dashed, grey line indicates the input distance (30 nm). Error bars are standard deviations.

3. The influence of false-positive signals

An uncertainty contribution that is difficult to quantify comes from the **data analysis** method. We have used software tools that are able to identify and analyse distances of DNA origami nanorulers automatically in a fast and usually satisfying way (e.g. with the GATTAnalysis software or the software package CAEOBS[3]). However, corresponding algorithms cannot be perfect as is illustrated in Figure S6. So, there is always a possibility of analysis artefacts leading to uncertainties that have to be taken into account when accurate measurements are needed. Filtering the results by hand can circumvent these artefacts, but can lead to uncertainties due to subjective picking of structures. We estimate the uncertainty contribution to be 1 nm. Here, this order of magnitude is estimated by the difference of both types of analysis (i.e. automatic and manual), which is (0.7≈1 nm). Furthermore, our analysis is based on picking signals within a superresolved image that are likely to be the result of a nanoruler because of a spot-to-spot distance within the expected range. However, there is also the unlikely possibility of e.g. two dimerized, broken nanorulers with one mark each in the expected distance range. This ends up with a non-zero possibility of picking such **false-positive structures**. Overall, we estimated the corresponding uncertainty contribution $u(\delta a_{fp})$ by the difference between the mean distance obtained by automatic and manual analysis of an exemplary SR-image: To avoid an underestimation of this influence we presume an uncertainty of 1.5 nm here, including potential uncertainty contributions form false-positive structures and subjective picking of structures. Since we have no information about the corresponding probability distribution, we estimate this value to be the interval of a rectangular distribution. This leads to a final uncertainty contribution of $1.5/\sqrt{3} \approx 0.9$ nm.

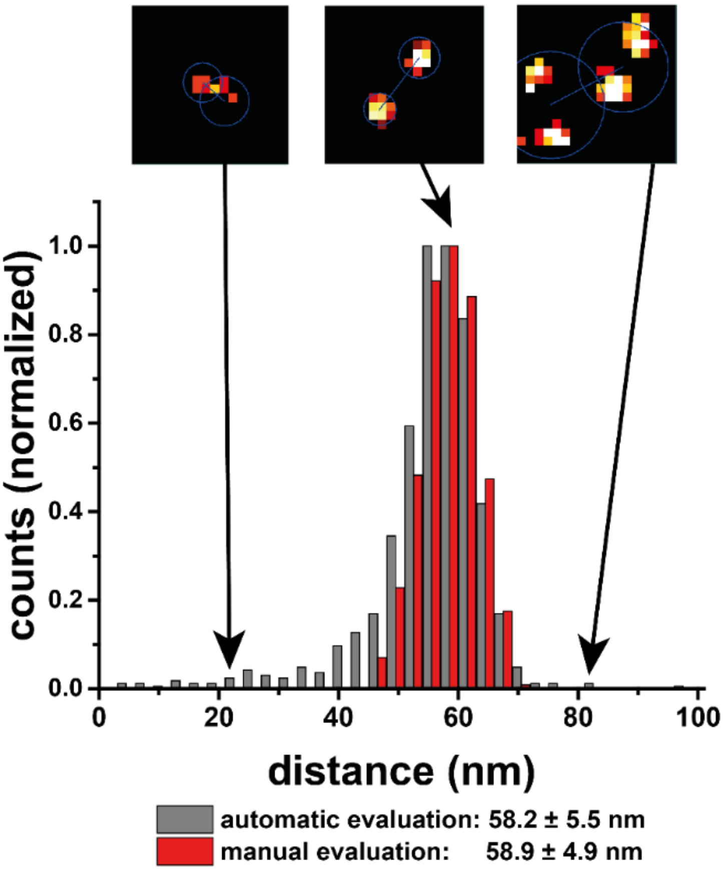

Figure S 6 Distance analysis with automatic and manual structure picking. The inset images show examples for a correct (middle) and wrong structures (left, right) picked by the automated analysis. Errors are standard deviations here.

4. The improved fit-model

Usually, distances in SR images are measured by fitting the cross section of two signals with the sum of two Gaussian functions in order to determine the difference between their centre. This procedure is however not efficient for DNA origami nanorulers: the need to project the structure on? one axis of the coordinate system takes a lot of time for large statistics and causes a systematic projection uncertainty in addition.

To circumvent this problem, we measured the distance between the two marks on a DNA origami by isolating their individual superresolved signals and histogramming the 2D-distances between all corresponding localizations like recently introduced[3]. This results in a bimodal distribution containing the distribution within the spots and between the spots that can be approximated by the function.

$$f(x) = c(x) + d(x) = ax \cdot \exp(-b \cdot x^2) + c \cdot \sqrt{x} \cdot \exp\left(-\frac{(x-x_0)^2}{2\sigma^2}\right)$$

The parameter x_0 represents the distance between the two marks. For its uncertainty determination two contributions have to be considered: the first one $u(\delta d_{fit})$ describes the uncertainty due to the fitting of the parameter (output of the LabView-routine “Nonlinear Curve Fit Intervals.vi”). This uncertainty contribution is stochastic and can

be decreased by averaging when the mean distance of a nanoruler ensemble is determined. On the other hand, the fit model is an approximation which results in a systematic uncertainty $u(\delta d_{model})$. Simulating two Gaussian distributed localization spots with a distance of a = 30 nm and different standard deviations (SD), we estimated that this uncertainty should not be larger than 0.5 nm as shown in Figure S7. However, note that the approximation only holds for s<<a. This means that this fit model is not suitable for the measurement of distances near the resolution limit. Because we have values of SD of about 5 nm in our measurements, it is a proper approximation in our case. Additionally compared to the fit model we have recently used[3,4] this model is improved by the factor $\sqrt{x}$. The old model is still a suitable approximation but with a systematic uncertainty of $\Delta a \approx s^2/a$. Under usual imaging condition this results in ($\Delta a \approx$ 0.5-1 nm) but can get worse at lower resolutions.

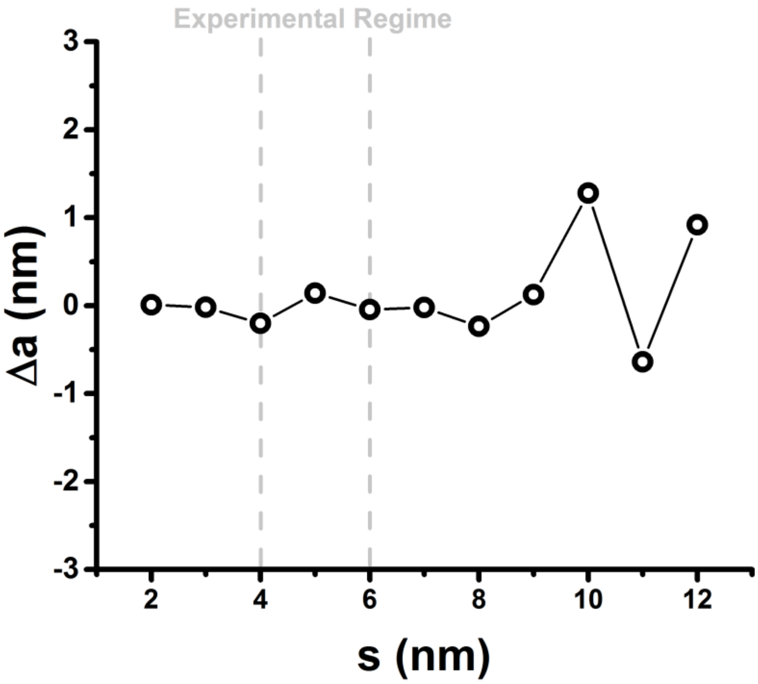

Figure S 7 Difference Δa between the measured and the simulated distance vs. the standard deviation s of the simulated Gaussian distributions. In the experimental regime Δa oscillates around 0 nm indicating that there is no significant systematic uncertainty within this interval.

5. Summary of different uncertainty contributions and their consideration in the measurement uncertainty budget.

As depicted in Figure 1e the development of the nanoruler distance uncertainty determination starts with an Ishikawa-diagram to visualize potential influences on the measurement. To make this development more transparent Table S5 provides a summary of these influences, their effect on the measurement and how they are considered with respect to the measurement uncertainty budget.

Table S 5 Summary of the possible uncertainty contributions from Figure 1e

Uncertainty contribution	Effect on the measurement	Consideration in Uncertainty budget
**Experimental setup uncertainties**		
**Pixelsize**		
Pixel size calibration uncertainty	Affects the numerical result	Considered in the uncertainty budget of the nanoruler calibration
Stage micrometre uncertainty	Affects the numerical result	Included in the pixel size calibration uncertainty
**Aberrations**		
Chromatic aberrations	Could affect distances of single nanorulers if occurrent	Not relevant: only one type of fluorophore (Atto655) is used in this calibration
Comatic aberrations	Makes signals asymmetrical	Neglected, not detectable
Field Curvature	Makes distance measurements position dependent	Included in the pixel size calibration uncertainty
**Environment**		
Temperature gradients	Defocussing due to changes of refractive index in the immersion oil or mechanic drift of stage components that leads to sample drift	Neglected: focus and temperature were stable during the measurements. Sample drift has been suppressed. The uncertainty of this suppression has however been considered. (see also “sample drift”)
Purity of immersion oil	Defocussing due to changes of refractive index in the immersion oil	Neglected: focus was stable during the measurements, oil was refreshed frequently
**Analysis uncertainties**		
Goodness of fits	Random uncertainty, acts on the uncertainty of single nanoruler distances	Considered in the uncertainty budget of the nanoruler calibration as random uncertainty
Evaluation model	Systematic deviations due to imperfectness of the mathematic model used for the analysis	Considered in the uncertainty budget of the nanoruler calibration as systematic uncertainty
Structure selection artefacts	Shift of the mean distance by false-positive results	Considered in the uncertainty budget of the nanoruler calibration
Imperfect automatic filters	Shift of the mean distance by false-positive results	Not relevant: data have been filtered manually for this calibration
**Sample uncertainties**		
**Emission properties**		
Brightness of dye	Limits the spot width and therefore the resolution: random uncertainty, can only lead to systematic uncertainties when it causes distances to be nearly unresolved.	Considered in the uncertainty budget of the nanoruler calibration as part of the overall random uncertainties.

Blinking kinetics	Can lead to systematic shortening of the distance at short off-times and long on-times	Systematic shortening is disabled due to the adjusted blinking kinetics by DNA-PAINT.<br>A small contribution is however considered in the uncertainty budget of the nanoruler calibration.
Photobleaching	Can limit the number of localizations	Not relevant since DNA-PAINT is used here.
**Structural defects of nanorulers**		
Missing staples	Missing staples in single DNA Origami structures can influence the distance of single nanorulers.<br>The probability is equal for every staple so the uncertainty should be random.	Considered in the uncertainty budget of the nanoruler calibration as part of the overall random uncertainties.
Non perfect-staples	Staples with false sequence can influence the distance of single nanorulers.<br>The probability is equal for every staple so the uncertainty should be random.	Considered in the uncertainty budget of the nanoruler calibration as part of the overall random uncertainties.
Labeling efficiency	Missing single labels can influence the distance of single nanorulers.<br>The probability is equal for every label so the uncertainty should be random.	Considered in the uncertainty budget of the nanoruler calibration as part of the overall random uncertainties.
**Imperfect geometry of nanorulers**		
Design simplifications	Simplifying assumptions during the nanoruler design lead to systematic differences between the nominal distance and the measured distance.	Not considered as an uncertainty: aim of the calibration is the proper determination of the actual distance.
3D-distortions	Distortions can occur from the DNA origami design, e.g. if a simple rectangular structure is not twist corrected.	Part of the reasons for the differences between nominal and measured distance: not considered as an uncertainty
Width of marks	Causes a slight broadening of the spots that leads to additional random uncertainties.	Considered in the uncertainty budget of the nanoruler calibration as part of the overall random uncertainties.
**Environmental influences**		
Sample drift	Sample drift can be caused by temperature gradients or other external influences and causes broadening of the spots. It has to be corrected or suppressed.	Drift has been suppressed to a non-detectable level for the calibration measurements. The uncertainty of this suppression has however been considered in the budget of the nanoruler calibration representing the result of environmental influences.
Immobilization strategy	Part of the chemical environment of the nanoruler and can have a systematic influence on the distance due to electrostatic forces.	Not relevant: calibration procedure holds for a single slide of nanorulers with a single immobilization strategy:<br>Influences are part of the difference between nominal and measured distance.
Buffer components	Considered in the uncertainty budget of the nanoruler calibration as part of the overall random uncertainties.<br>Especially salt concentrations can have an influence due to the interaction of cations with the DNA backbone.	Not relevant: calibration procedure holds for a single slide of nanorulers with a single immobilization strategy:<br>Influences are part of the difference between nominal and measured distance.

6. Exemplary drift correction for comparison with Figure 4

As mentioned above, drift can be corrected by tracking the signal of fiducial markers over time. This is most commonly done in single-molecule localization microscopy (SMLM) while drift suppression like shown in Figure 4 of the main text is rather unusual. To give a comparison for the same sample we show some data here where drift is not suppressed but corrected.

We use a commercial product, namely GATTAquant HiRes beacons made for DNA-PAINT and the corresponding analysis software (GATTAnalysis by GATTAquant GmbH). As illustrated in Figure S8. localization positions of the fiducial markers are plotted over time. Then a polynomic of desired order or a spline (sliding average) is fitted to the data points in order to calculate a proper correction function. If the nanoruler spots do not show a detectable asymmetry after drift correction, possible resulting uncertainties resulting from drift can be neglected.

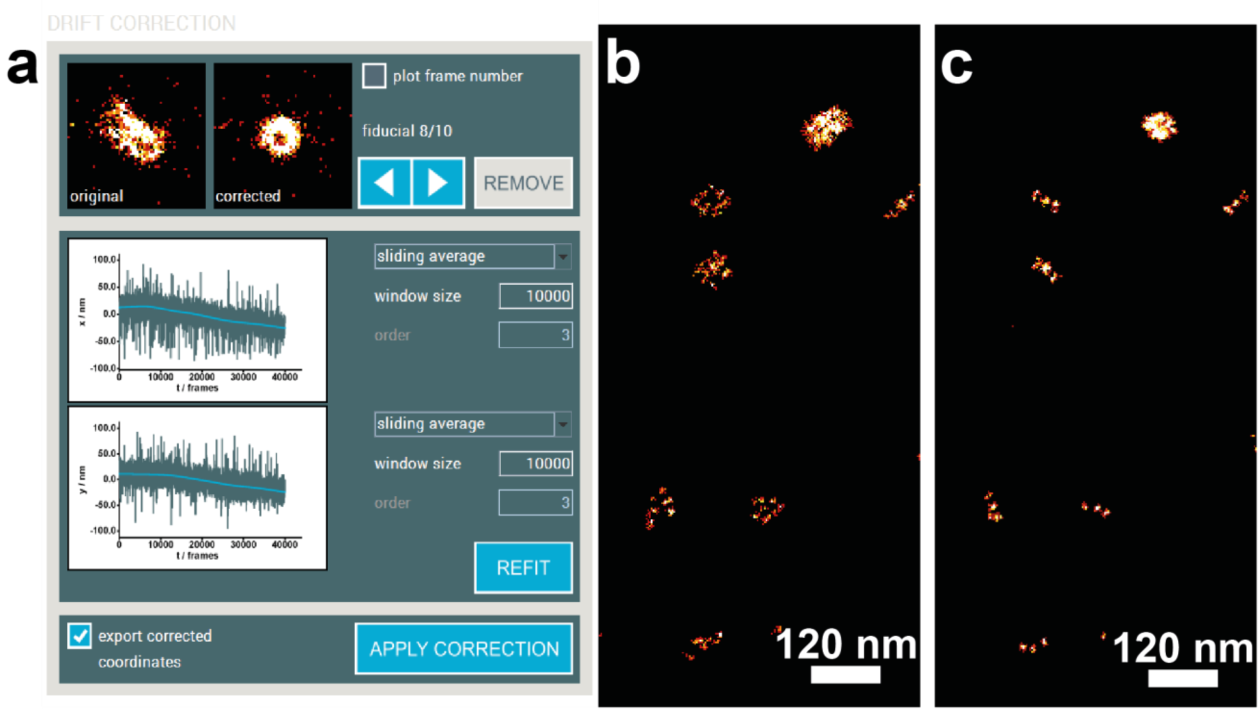

Figure S 8 Example for drift correction after image acquisition using GATTA-PAINT HiRes 20R nanorulers. a) Graphical user interface of the GATTAnalysis software. b) SR-image without drift correction. c) The same image after drift correction.

7. Correction of chromatic aberrations

As described in the manuscript we use the following equation system to transfer the coordinates of the green colour channel into the coordinates of the red colour channel in order to correct chromatic aberrations.

$$x_{red}(x,y) = a \cdot x_{green} + b \cdot y_{green} + c$$

$$y_{red}(x,y) = d \cdot x_{green} + e \cdot y_{green} + f$$

Both equations have the same general form:

$$f(x, y) = ax + by + c$$

Such an equation can be solved analytically with help of multicolour-bead data by minimizing the squared difference S from the red reference coordinates f_i and the function $f(x_i, y_i)$. The beads give the coordinates x_i, y_i.

$$S = \sum_i \left(f_i - f(x_i, y_i)\right)^2 = \sum_i (f_i - ax_i - by_i - c)^2$$

$$\frac{d}{da}S = \sum_i -2x_i(f_i - ax_i - by_i - c) = 0$$

$$\frac{d}{db}S = \sum_i -2y_j(f_i - ax_i - by_i - c) = 0$$

$$\frac{d}{dc}S = \sum_i -2(f_i - ax_i - by_i - c) = 0$$

$$\sum_i (-2x_i f_i + 2x_i^2 a + 2x_i y_i b + 2x_i c) = 0$$

$$\sum_i (-2y_i f_i + 2x_i y_i a + 2y_i^2 b + 2y_i c) = 0$$

$$\sum_i (-2f_i + 2x_i a + 2y_i b + 2c) = 0$$

This yields the following equation system:

$$\left(\sum_i (-2x_i f_i)\right) + \left(\sum_i (2x_i^2)\right) a + \left(\sum_i (2x_i y_i)\right) b + \left(\sum_i (2x_i)\right) c = 0$$

$$\left(\sum_i (-2y_i f_i)\right) + \left(\sum_i (2x_i y_i)\right) a + \left(\sum_i (2y_i^2)\right) b + \left(\sum_i (2y_i)\right) c = 0$$

$$\left(\sum_i (-2f_i)\right) + \left(\sum_i (2x_i)\right) a + \left(\sum_i (2y_i)\right) b + \left(\sum_i (2)\right) c = 0$$

Solving this equation system for both initial equations yields the correction parameters a, b and c, as well as d, e, and f that can be used to correct the green coordinates in order to merge them with the red ones. As seen in Figure 3c, d, f this procedure gives a proper correction.

8. DNA Sequences for custom DNA origami nanorulers

Imager strands:
CGGGCATT-Atto655 (8 bases)
CGGGCA-Atto655 (6 bases)

Table S 6 Sequences of modified DNA-staples used for rectangular origami with designed distances of 32.5 and 65 nm.

Number	**Sequence (5‘ - 3‘)**
	**Biotin-strands**
1	Biotin-GAGAAGAGATAACCTTGCTTCTGTTCGGGAGAAACAATAA
2	Biotin-<br>GAAACGATAGAAGGCTTATCCGGTCTCATCGAGAACAAGC
3	Biotin-<br>CGGATTCTGACGACAGTATCGGCCGCAAGGCGATTAAGTT
4	Biotin-<br>AGCCACCACTGTAGCGCGTTTTCAAGGGAGGGAAGGTAAA
5	Biotin-<br>ATAAGGGAACCGGATATTCATTACGTCAGGACGTTGGGAA
6	Biotin-TAGAGAGTTATTTTCATTTGGGGATAGTAGTAGCATTA
	**DNA-PAINT docking strands used for 65 nm and 32.5 nm**
7	GATTTAGTCAATAAAGCCTCAGAGAACCCTCATTAAATGCCCG
8	AAATTAAGTTGACCATTAGATACTTTTGCGTTAAATGCCCG
9	ATGCAGATACATAACGGGAATCGTCATAAATAAAGCAAAGTTAA<br>ATGCCCG
10	CGGATTGCAGAGCTTAATTGCTGAAACGAGTATTAAATGCCCG
11	GATGGCTTATCAAAAAGATTAAGAGCGTCCTTAAATGCCCG
12	GCTATCAGAAATGCAATGCCTGAATTAGCATTAAATGCCCG
13	AACAAGAGGGATAAAAATTTTTAGCATAAAGCTTAAATGCCCG
14	TAAATCGGGATTCCCAATTCTGCGATATAATGTTAAATGCCCG
15	CTGTAGCTTGACTATTATAGTCAGTTCATTGATTAAATGCCCG
	**DNA-PAINT docking strands used only for 32.5 nm**
16	CCAATAGCTCATCGTAGGAATCATGGCATCAATTAAATGCCCG
17	TTAACGTCTAACATAAAAACAGGTAACGGATTAAATGCCCG
18	AGAGAGAAAAAAATGAAAATAGCAAGCAAACTTTAAATGCCCG
19	CAACCGTTTCAAATCACCATCAATTCGAGCCATTAAATGCCCG
20	CATGTAATAGAATATAAAGTACCAAGCCGTTTAAATGCCCG
21	CCAACAGGAGCGAACCAGACCGGAGCCTTTACTTAAATGCCCG
22	TTCTACTACGCGAGCTGAAAAGGTTACCGCGCTTAAATGCCCG
23	TTTTATTTAAGCAAATCAGATATTTTTTGTTTAAATGCCCG
24	TTATTACGAAGAACTGGCATGATTGCGAGAGGTTAAATGCCCG

	DNA-PAINT docking strands used for 65nm
25	GAAACGATAGAAGGCTTATCCGGTCTCATCGAGAACAAGCTTAAATGCCCG
26	CTTATCATTCCCGACTTGCGGGAGCCTAATTTTTAAATGCCCG
27	GCCAGTTAGAGGGTAATTGAGCGCTTTAAGAATTAAATGCCCG
28	GTTTATTTTGTCACAATCTTACCGAAGCCCTTTAATATCATTAAATGCCCG
29	TTAGTATCACAATAGATAAGTCCACGAGCATTAAATGCCCG
30	TGTAGAAATCAAGATTAGTTGCTCTTACCATTAAATGCCCG
31	GAGAGATAGAGCGTCTTTCCAGAGGTTTTGAATTAAATGCCCG
32	ACGCTAACACCCACAAGAATTGAAAATAGCTTAAATGCCCG
33	GCCTTAAACCAATCAATAATCGGCACGCGCCTTTAAATGCCCG

Table S 7 Sequences of unmodified DNA-staples used for rectangular origami with designed distances of 32.5 and 65 nm.

Number	**Sequence (5‘ - 3‘)**
34	CATAAATCTTTGAATACCAAGTGTTAGAAC
35	GATGTGCTTCAGGAAGATCGCACAATGTGA
36	GCAATTCACATATTCCTGATTATCAAAGTGTA
37	TCACCAGTACAAACTACAACGCCTAGTACCAG
38	GCTTTCCGATTACGCCAGCTGGCGGCTGTTTC
39	AAAGGCCGGAGACAGCTAGCTGATAAATTAATTTTTGT
40	AAGCCTGGTACGAGCCGGAAGCATAGATGATG
41	TCATTCAGATGCGATTTTAAGAACAGGCATAG
42	GCCATCAAGCTCATTTTTTAACCACAAATCCA
43	TATAACTAACAAAGAACGCGAGAACGCCAA
44	TTGCTCCTTTCAAATATCGCGTTTGAGGGGGT
45	GTATAGCAAACAGTTAATGCCCAATCCTCA
46	AAAGTCACAAAATAAACAGCCAGCGTTTTA
47	GGCCTTGAAGAGCCACCACCCTCAGAAACCAT
48	AGTATAAAGTTCAGCTAATGCAGATGTCTTTC
49	TCAAATATAACCTCCGGCTTAGGTAACAATTT
50	TTTCGGAAGTGCCGTCGAGAGGGTGAGTTTCG
51	GAGGGTAGGATTCAAAAGGGTGAGACATCCAA
52	TATTAAGAAGCGGGGTTTTGCTCGTAGCAT
53	GCCCTTCAGAGTCCACTATTAAAGGGTGCCGT
54	AGCCAGCAATTGAGGAAGGTTATCATCATTTT
55	TAAATGAATTTTCTGTATGGGATTAATTTCTT
56	AAACAGCTTTTTGCGGGATCGTCAACACTAAA
57	GCGCAGACAAGAGGCAAAAGAATCCCTCAG
58	GACAAAAGGTAAAGTAATCGCCATATTTAACAAAACTTTT
59	ACACTCATCCATGTTACTTAGCCGAAAGCTGC
60	CTACCATAGTTTGAGTAACATTTAAAATAT
61	TATATTTTGTCATTGCCTGAGAGTGGAAGATTGTATAAGC
62	TAAATCATATAACCTGTTTAGCTAACCTTTAA
63	GTACCGCAATTCTAAGAACGCGAGTATTATTT

64	TCTTCGCTGCACCGCTTCTGGTGCGGCCTTCC
65	GCAAGGCCTCACCAGTAGCACCATGGGCTTGA
66	ATTACCTTTGAATAAGGCTTGCCCAAATCCGC
67	CTTATCATTCCCGACTTGCGGGAGCCTAATTT
68	TTATACCACCAAATCAACGTAACGAACGAG
69	GTAATAAGTTAGGCAGAGGCATTTATGATATT
70	GATGGTTTGAACGAGTAGTAAATTTACCATTA
71	GCACAGACAATATTTTTGAATGGGGTCAGTA
72	AGCAAGCGTAGGGTTGAGTGTTGTAGGGAGCC
73	TCCACAGACAGCCCTCATAGTTAGCGTAACGA
74	ATTATACTAAGAAACCACCAGAAGTCAACAGT
75	TAAGAGCAAATGTTTAGACTGGATAGGAAGCC
76	ATACATACCGAGGAAACGCAATAAGAAGCGCATTAGACGG
77	CAACTGTTGCGCCATTCGCCATTCAAACATCA
78	TAGGTAAACTATTTTTGAGAGATCAAACGTTA
79	AGGCAAAGGGAAGGGCGATCGGCAATTCCA
80	ATTATCATTCAATATAATCCTGACAATTAC
81	GAAATTATTGCCTTTAGCGTCAGACCGGAACC
82	AATGGTCAACAGGCAAGGCAAAGAGTAATGTG
83	ATACCCAACAGTATGTTAGCAAATTAGAGC
84	CACCAGAAAGGTTGAGGCAGGTCATGAAAG
85	ATCCCAATGAGAATTAACTGAACAGTTACCAG
86	GACCTGCTCTTTGACCCCCAGCGAGGGAGTTA
87	AGGAACCCATGTACCGTAACACTTGATATAA
88	CAGCGAAACTTGCTTTCGAGGTGTTGCTAA
89	ACAACTTTCAACAGTTTCAGCGGATGTATCGG
90	CAGCAAAAGGAAACGTCACCAATGAGCCGC
91	ACCTTTTTATTTTAGTTAATTTCATAGGGCTT
92	CGATAGCATTGAGCCATTTGGGAACGTAGAAA
93	GCCCGAGAGTCCACGCTGGTTTGCAGCTAACT
94	ATTTTAAAATCAAAATTATTTGCACGGATTCG
95	ACCTTGCTTGGTCAGTTGGCAAAGAGCGGA
96	CTGAGCAAAAATTAATTACATTTTGGGTTA
97	CCTGATTGCAATATATGTGAGTGATCAATAGT
98	TCAATATCGAACCTCAAATATCAATTCCGAAA
99	CTTTAGGGCCTGCAACAGTGCCAATACGTG
100	AATAGTAAACACTATCATAACCCTCATTGTGA
101	TCACCGACGCACCGTAATCAGTAGCAGAACCG
102	GCCCGTATCCGGAATAGGTGTATCAGCCCAAT
103	TGTAGCCATTAAAATTCGCATTAAATGCCGGA
104	TCGGCAAATCCTGTTTGATGGTGGACCCTCAA
105	TGACAACTCGCTGAGGCTTGCATTATACCA
106	CCACCCTCTATTCACAAACAAATACCTGCCTA
107	CCCGATTTAGAGCTTGACGGGGAAAAAGAATA
108	AAGTAAGCAGACACCACGGAATAATATTGACG
109	CACATTAAAATTGTTATCCGCTCATGCGGGCC
110	TTAAAGCCAGAGCCGCCACCCTCGACAGAA
111	ATATTCGGAACCATCGCCCACGCAGAGAAGGA

112	AACGTGGCGAGAAAGGAAGGGAAACCAGTAA
113	GAATTTATTTAATGGTTTGAAATATTCTTACC
114	AGCGCGATGATAAATTGTGTCGTGACGAGA
115	AACGCAAAGATAGCCGAACAAACCCTGAAC
116	GCCTCCCTCAGAATGGAAAGCGCAGTAACAGT
117	AAAGCACTAAATCGGAACCCTAATCCAGTT
118	AAGGCCGCTGATACCGATAGTTGCGACGTTAG
119	CTTTTGCAGATAAAAACCAAAATAAAGACTCC
120	CCTAAATCAAAATCATAGGTCTAAACAGTA
121	AGACGACAAAGAAGTTTTGCCATAATTCGAGCTTCAA
122	AGAAAACAAAGAAGATGATGAAACAGGCTGCG
123	CGCGCAGATTACCTTTTTAATGGGAGAGACT
124	CACAACAGGTGCCTAATGAGTGCCCAGCAG
125	GCGGAACATCTGAATAATGGAAGGTACAAAAT
126	TAAAAGGGACATTCTGGCCAACAAAGCATC
127	AATTGAGAATTCTGTCCAGACGACTAAACCAA
128	GCGAAAAATCCCTTATAAATCAAGCCGGCG
129	AACACCAAATTTCAACTTTAATCGTTTACC
130	TAAATCAAAATAATTCGCGTCTCGGAAACC
131	GCGAACCTCCAAGAACGGGTATGACAATAA
132	TTAGGATTGGCTGAGACTCCTCAATAACCGAT
133	ATCGCAAGTATGTAAATGCTGATGATAGGAAC
134	GCGGATAACCTATTATTCTGAAACAGACGATT
135	AAGGAAACATAAAGGTGGCAACATTATCACCG
136	ACCCTTCTGACCTGAAAGCGTAAGACGCTGAG
137	ATATTTTGGCTTTCATCAACATTATCCAGCCA
138	TCAAGTTTCATTAAAGGTGAATATAAAAGA
139	TCTAAAGTTTTGTCGTCTTTCCAGCCGACAA
140	TTCCAGTCGTAATCATGGTCATAAAAGGGG
141	AATACTGCCCAAAAGGAATTACGTGGCTCA
142	TTTATCAGGACAGCATCGGAACGACACCAACCTAAAACGA
143	TTGACAGGCCACCACCAGAGCCGCGATTTGTA
144	CTGTGTGATTGCGTTGCGCTCACTAGAGTTGC
145	GCGAGTAAAAATATTTAAATTGTTACAAAG
146	CGAAAGACTTTGATAAGAGGTCATATTTCGCA
147	TCATCGCCAACAAAGTACAACGGACGCCAGCA
148	TTAACACCAGCACTAACAACTAATCGTTATTA
149	ACAACATGCCAACGCTCAACAGTCTTCTGA
150	CATTTGAAGGCGAATTATTCATTTTTGTTTGG
151	TGAAAGGAGCAAATGAAAAATCTAGAGATAGA
152	TGGAACAACCGCCTGGCCCTGAGGCCCGCT
153	TACCGAGCTCGAATTCGGGAAACCTGTCGTGCAGCTGATT
154	ACAAACGGAAAAGCCCCAAAAACACTGGAGCA
155	GTTTATCAATATGCGTTATACAAACCGACCGTGTGATAAA
156	ACGGCTACAAAAGGAGCCTTTAATGTGAGAAT
157	GACCAACTAATGCCACTACGAAGGGGGTAGCA
158	CTCCAACGCAGTGAGACGGGCAACCAGCTGCA
159	ACCGATTGTCGGCATTTTCGGTCATAATCA
160	CAGAAGATTAGATAATACATTTGTCGACAA

161	TGCATCTTTCCCAGTCACGACGGCCTGCAG
162	GTTTTAACTTAGTACCGCCACCCAGAGCCA
163	TTAATGAACTAGAGGATCCCCGGGGGGTAACG
164	CTTTTACAAAATCGTCGCTATTAGCGATAG
165	ATCCCCCTATACCACATTCAACTAGAAAAATC
166	AGAAAGGAACAACTAAAGGAATTCAAAAAAA
167	GCCGTCAAAAAACAGAGGTGAGGCCTATTAGT
168	CCACCCTCATTTTCAGGGATAGCAACCGTACT
169	CTTTAATGCGCGAACTGATAGCCCCACCAG
170	CCAGGGTTGCCAGTTTGAGGGGACCCGTGGGA
171	CAAATCAAGTTTTTTGGGGTCGAAACGTGGA
172	TACGTTAAAGTAATCTTGACAAGAACCGAACT
173	TAATCAGCGGATTGACCGTAATCGTAACCG
174	TTTTCACTCAAAGGGCGAAAAACCATCACC
175	AATAGCTATCAATAGAAAATTCAACATTCA
176	CATCAAGTAAAACGAACTAACGAGTTGAGA
177	CAGGAGGTGGGGTCAGTGCCTTGAGTCTCTGAATTTACCG
178	AAATCACCTTCCAGTAAGCGTCAGTAATAA
179	CTCGTATTAGAAATTGCGTAGATACAGTAC
180	TTTACCCCAACATGTTTTAAATTTCCATAT
181	GTCGACTTCGGCCAACGCGCGGGGTTTTTC
182	CGTAAAACAGAAATAAAAATCCTTTGCCCGAAAGATTAGA
183	AGGCTCCAGAGGCTTTGAGGACACGGGTAA
184	TTTAGGACAAATGCTTTAAACAATCAGGTC
185	AATACGTTTGAAAGAGGACAGACTGACCTT
186	CTTAGATTTAAGGCGTTAAATAAAGCCTGT
187	AACAGTTTTGTACCAAAAACATTTTATTTC
188	AACGCAAAATCGATGAACGGTACCGGTTGA

References

1. BIPM, IEC, IFCC, ILAC, ISO, IUPAC, IUPAP, OIML. Guide to the expression of uncertainty in measurement - JCGM 100:2008 (GUM 1995 with minor corrections - Evaluation of measurement data **2008** (2008).
2. Jungmann, R. *et al.* Single-Molecule Kinetics and Super-Resolution Microscopy by Fluorescence Imaging of Transient Binding on DNA Origami. *Nano letters* **10,** 4756–4761 (2010).
3. Schmied, J. J. *et al.* DNA origami-based standards for quantitative fluorescence microscopy. *Nat Protoc* **9,** 1367–1391 (2014).
4. Schmied, J. J. *et al.* Fluorescence and super-resolution standards based on DNA origami. *Nat Methods* **9,** 1133–1134 (2012).
